# Influence of Carbohydrate Intake on Different Parameters of Soccer Players’ Performance: Systematic Review

**DOI:** 10.3390/nu16213731

**Published:** 2024-10-31

**Authors:** Marián Pueyo, Iñaki Llodio, Jesús Cámara, Daniel Castillo, Cristina Granados

**Affiliations:** 1 Faculty of Education and Sport, University of the Basque Country, UPV/EHU, 01007 Vitoria-Gasteiz, Spain; mpueyo003@ikasle.ehu.eus (M.P.); inaki.llodio@ehu.eus (I.L.); jesus.camara@ehu.eus (J.C.); cristina.granados@ehu.eus (C.G.); 2 Society, Sports and Physical Exercise Research Group (GIKAFIT), Department of Physical Education and Sport, Faculty of Education and Sport, University of the Basque Country (UPV/EHU), 01007 Vitoria-Gasteiz, Spain; 3 AKTIBOki, Research Group in Physical Activity, Physical Exercise and Sport, Department of Physical Education and Sport, Faculty of Education and Sport, University of the Basque Country (UPV/EHU), 01007 Vitoria-Gasteiz, Spain; 4 Valoración del Rendimiento Deportivo, Actividad Física y Salud y Lesiones Deportivas (REDAFLED), Department of Didactics of Musical, Plastic and Corporal Expression, Faculty of Education, University of Valladolid, 42004 Soria, Spain

**Keywords:** carbohydrates, soccer, nutrition, muscle recovery, performance

## Abstract

Background: The objective of this systematic review is to analyze the influence of carbohydrate (CHO) intake on physical and technical aspects, glucose and muscle glycogen levels, fatigue, cognition, and gastrointestinal comfort involved in the performance of soccer players, as well as to examine whether there are any differences between men and women. Methods: A bibliographic search was conducted in PubMed, Web of Science, Scopus, and SportDiscus, resulting in 61 selected articles. The PRISMA recommendations and the Cochrane Handbook for Systematic Reviews guidelines were followed. Results: The results indicate that CHO intake before and during the match improves speed and the number of sprints, attenuates the decrease in shooting accuracy and speed, increases time to fatigue, and enhances cognitive function. There is no consensus on passing, dribbling, jumping, or agility improvements. Glucose levels drop during the first 15 min of the second half without affecting performance. Conclusions: It is recommended that players ingest 6–8 g/kg/d of CHO the day before, a meal with 1–3 g/kg 3–4 h before, and 30–60 g/h during the match. Muscle glycogen drops drastically at the end of the match, remaining low at 48 h. Hence, 1–1.5 g/kg/h is recommended during the first 4 h, starting from the first 20 min. Female soccer players have a similar physical demand to men, and energy availability is low, especially in the post-match periods, as they underestimate their energy expenditure and do not consume enough CHO. Therefore, the recommended guidelines should be followed, individualized, and periodized according to each athlete’s energy needs.

## 1. Introduction

### 1.1. Physical Demands in Soccer

Soccer is a constantly evolving team sport. In recent years, physical and technical demands have increased [1,2,3], along with continuous tactical modifications, undoubtedly influenced by the economic implications of winning or losing. Consequently, the demands of training have risen, becoming increasingly sophisticated to condition players and meet these demands throughout the season [4,5,6]. Players must be prepared to run at high speeds, have endurance and agility, jump, head the ball, pass, shoot, accelerate, decelerate, etc., all while attempting to mitigate the wear and injuries associated with the current accumulation of matches [1,2,6,7,8]. This sport is thus characterized by an intermittent activity profile, combining high and low-intensity actions (10% and 90%, respectively) [8,9].

Although in recent decades the total distance covered in kilometers has decreased by around 2%, the number of high-intensity actions has increased by 30–50% and the number of sprints by 85% [4]. On average, players typically cover between 10–14 km per game, with more than 8% at high intensity, >85–90% of maximum heart rate for 40% of the total game time, and an average oxygen consumption of around 70% of maximum oxygen consumption [1,2,8,10,11]. This scenario presents the challenge of requiring optimal recovery, dietary, and rest strategies that meet the energy expenditure demands, optimizing energy reserves, alleviating fatigue, maintaining optimal body mass, improving sports performance, preventing injuries and overtraining symptoms, and promoting rapid recovery [1,2,3,8]. Physical and mental fatigue negatively affects performance, resulting from glycogen depletion, dehydration, and muscle damage, with contributing intrinsic (age, sex, body composition, etc.) and extrinsic factors (result, quality of the opponent, location, playing surface, etc.) [2,3,8,12,13,14].

### 1.2. Nutrition: A Fundamental Pillar of Soccer

The field of sports nutrition has seen significant evolution in recent years, with ongoing research continually uncovering new insights. This dynamic nature of sports nutrition is crucial, as it decisively influences soccer players’ preparation, performance, and recovery during and between matches, ensuring their physical and mental well-being [3,4,5,7]. However, the evolving nature of this field can also lead to confusion, especially in the face of marketing influences, making it challenging for coaches, physical trainers, parents, and players—many of whom lack nutritional knowledge and education—to interpret the findings [8]. Therefore, it is essential to educate all these parties about the importance of proper nutrition for physical and mental health, as well as performance outcomes [8,15]. The primary goal should be to ensure effective nutritional support in main meals (type of nutrients, quantity, timing, method of administration, etc.), which can be supplemented with carbohydrate (CHO)-rich supplements—drinks, gels, or snacks—aimed at achieving certain health, performance, and/or recovery benefits, considering the individual and personalized response of each player according to their physiology, possible gastrointestinal discomfort according to the type and volume of CHO, the periodization, volume, and intensity of training, the phase of the season, and their playing position [2,8,16].

The challenge in soccer is addressing specific contexts and individualizing training and nutrition. The schedules involve a multitude of timings, travel, geographical areas, and climates, to which must be added the various nationalities that make up a team, with very different cultures and eating habits, potentially leading to decreased appetite and changes in food preferences [8].

Intake can have a profound impact on body composition. During the season, there may be times when the goal is to manipulate macronutrient intake to rapidly alter aspects such as muscle mass or fat mass, something common in pre-season or during injury recovery. However, this must be well-justified, thoroughly planned, and executed with the player’s full agreement [11]. Diet manipulation focused on reducing CHO intake can adversely affect performance [2,3], not to mention that body composition depends on the player’s physiology, position, and style of play.

Therefore, it is easy to understand that nutrition in soccer must be personalized, monitored, and supervised according to individualized goals, must align with physical objectives, and must be directed by a nutritional team [2,8].

### 1.3. Nutrition Female Soccer Players

Decades of research on the physiological demands of soccer have primarily focused on men, resulting in a limitation of studies analyzing dietary intake and nutritional strategies for female soccer players [10,15]. Nutritional guidelines for female soccer players are often based on research conducted on males. However, it is known that many female players are “under-fueled” and do not consume sufficient energy to match their expenditure [10,15]. This can be attributed to a lack of knowledge about recommended nutritional guidelines, the absence of accredited nutrition personnel, or misunderstandings about the impact of CHO intake on body image, influenced by external pressures (parents, coaches, etc.), social media, and stereotypes surrounding body composition measurements [15].

There is often no correlation between energy needs and appetite in female soccer players. In fact, a high prevalence of low energy availability (EA)—the total dietary energy resulting from the difference between energy intake and energy expenditure induced by exercise, measured in kcal/kg of body mass—is commonly found during periods of intense training, associated with a lack of compensatory appetite. This can result in symptoms associated with the “female athlete triad,” which includes hormonal disturbances, menstrual dysfunctions, and impaired bone health [10,15,17]. This occurs when EA falls below 30 kcal/kg/day, and the symptoms are often attributed to exercise rather than low EA [18]. Therefore, the minimum energy requirement has been set at 45 kcal/kg/day to meet athletes’ minimal needs [10,19]. Additionally, it is essential to understand that EA varies daily based on training, matches, position, and style of play. However, nowadays, there is a greater focus on body mass than energy requirements, estimating energy expenditure and calculating the necessary energy intake based on player movement [20,21].

### 1.4. Carbohydrates: The Primary Energy Source for Muscles

Carbohydrates are a primary muscle fuel source and are also utilized by the central nervous system during sports activities, especially at high intensities [1,3,8,20,22,23]. They constitute the key macronutrient for match preparation, providing 60–70% of the energy needed on match day [8,10]. The role of muscle glycogen during matches has been extensively studied [6], but less so during training sessions, which vary systematically based on the upcoming match’s demands. Muscle and liver glycogen depletion is a limiting factor for performance [1], and CHO intake is crucial to ensure optimal glycogen reserves and maximize performance [2,3,8,10].

After a match, both men and women may find their glycogen stores nearly or completely empty in up to 80% of type I muscle fibers and 70% of type II fibers. Even after 48 h, complete restoration of type II fibers is not evident, often accompanied by muscular pain and impairment lasting up to 72 h [1,3,6,10,11,24]. Muscle glycogen depletion can reach 50% after matches and about 20% after training sessions [1,11]. Up to 50% of liver glycogen can be depleted after an overnight fast and may not fully recover until the early evening hours [1,10].

Historically, invasive muscle biopsies were used to assess skeletal muscle glycogen reserves. However, recently, a novel high-frequency skeletal muscle ultrasound methodology has been developed and validated, providing rapid, portable, and non-invasive results [25]. Despite players adjusting CHO intake relative to training intensity and volume, their intake often falls below recommended levels for both sexes and across all age groups during training sessions, matches (pre-, during, and post), rest days, pre-season, and regular season, occasionally compensating by exceeding protein intake recommendations [1,2,3,8,10,17,26,27,28].

During high-intensity actions such as sprints, anaerobic energy production primarily relies on intramuscular glycogen and phosphocreatine stores. In soccer, with frequent changes in pace, energy derived from aerobic glycogen degradation is slow and insufficient during those moments, crucial for recovery during periods of low intensity and increasingly important as the match progresses. Beyond quantity, CHO’s quality and glycemic index (GI) are also significant [8].

Thus, the recommended intake of high-GI CHO with low protein content, consumed 4 h before bedtime, reduces sleep onset latency more effectively than intake 1 h before, although further data is needed on its performance influence [29]. However, the scientific literature lacks comprehensive studies combining the relevance of CHO intake on soccer performance across genders, all age categories, physical and technical aspects parameters, glucose and muscle glycogen levels, fatigue, energy availability, and cognition. Moreover, there is a dearth of studies describing habitual CHO intake practices in this population and presenting basic intake recommendations for this macronutrient.

### 1.5. Objective

This systematic review aimed to analyze the influence of CHO intake on physical and technical aspects, glucose and muscle glycogen levels, fatigue, energy availability, and cognition in soccer players’ performance and examine any differences between men and women.

## 2. Methods

### 2.1. Design

This systematic review was conducted using the PRISMA recommendations [30] and the guidelines of the Cochrane Handbook for Systematic Reviews [31]. The protocol was registered and published in PROSPERO (ID = CRD42024537954).

### 2.2. Search Strategy

A systematic search was conducted in PubMed, Scopus, Web of Science, and SportDiscus databases between 10 and 20 December 2023. The search and selection criteria were designed to obtain high-quality, up-to-date material, encompassing all studies published from 2010 to 2023.

To ensure the inclusion of all relevant information, a comprehensive set of search criteria was developed using the following keyword combinations: carbohydrate AND (soccer OR football) AND ((diet* OR nutr* OR macronutrient OR energy expenditure OR energy intake) OR (performance OR exercis* OR fitness OR capacit*) OR (female OR women)). The search combinations were entered as follows: “all fields” for PubMed; “article title, abstract, keywords” for Scopus; boolean/phrase and apply equivalent subjects (Expanders) for SportDiscus; and “topic” for Web of Science. Articles in English or Spanish were included.

### 2.3. Inclusion and Exclusion Criteria

All peer-reviewed articles in English published online were considered, regardless of the publication status. Experimental and descriptive observational articles on soccer players of any category, level, and age range were included. Reviews, commentaries, editorials, letters, and meeting abstracts were excluded. Articles exclusively involving rugby, American football, Australian football, or Gaelic football players were not included. Only studies that analyzed the influence of CHO intake on physical and technical aspects, glucose and muscle glycogen levels, fatigue, energy availability, and cognition were included in the review.

### 2.4. Study Selection

Two authors (M.P. and C.G.) independently performed the search, and all identified records exported from the databases were imported to EndNote 20.3 (Clarivate Analytics, Philadelphia, PA, USA). Duplicate records were identified using the “Find duplicates” function of EndNote and manually removed after a manual check by M.P. and C.G. Screening of title and abstract was conducted separately by M.P. and C.G., and in case of uncertainty, the full-text article was checked for verification. Disagreement regarding inclusion was discussed by M.P. and C.G.

**Figure 1:**
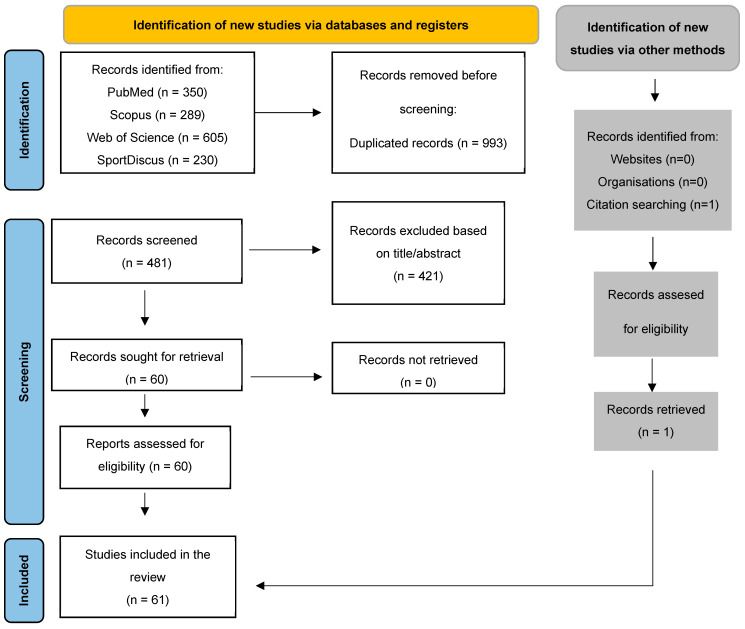
PRISMA flow chart.

The database search identified 1474 records. After removing the duplicates, 993 records were excluded. After screening the titles and abstracts, 421 records were excluded. The full text of the 60 reports was assessed for eligibility. Additionally, one record was added using other methods. In total, 61 studies, 34 experimental and 27 descriptive trials, involving a total sample of 1111 adult soccer players (659 men and 452 women), met the inclusion criteria and were extracted for qualitative analysis. Study identification, screening, and selection process using the PRISMA flow diagram is illustrated in Figure 1.

### 2.5. Data Extraction

One author (M.P.) extracted data on the population (i.e., number of players, competitive level, sex, and age), country of origin, supplementation protocol (i.e., substance or nutrient, dose, and duration), and performance outcomes from the included studies and subsequently reviewed them by another author (M.P. and C.G.).

### 2.6. Risk of Bias of the Studies

**Figure 2:**
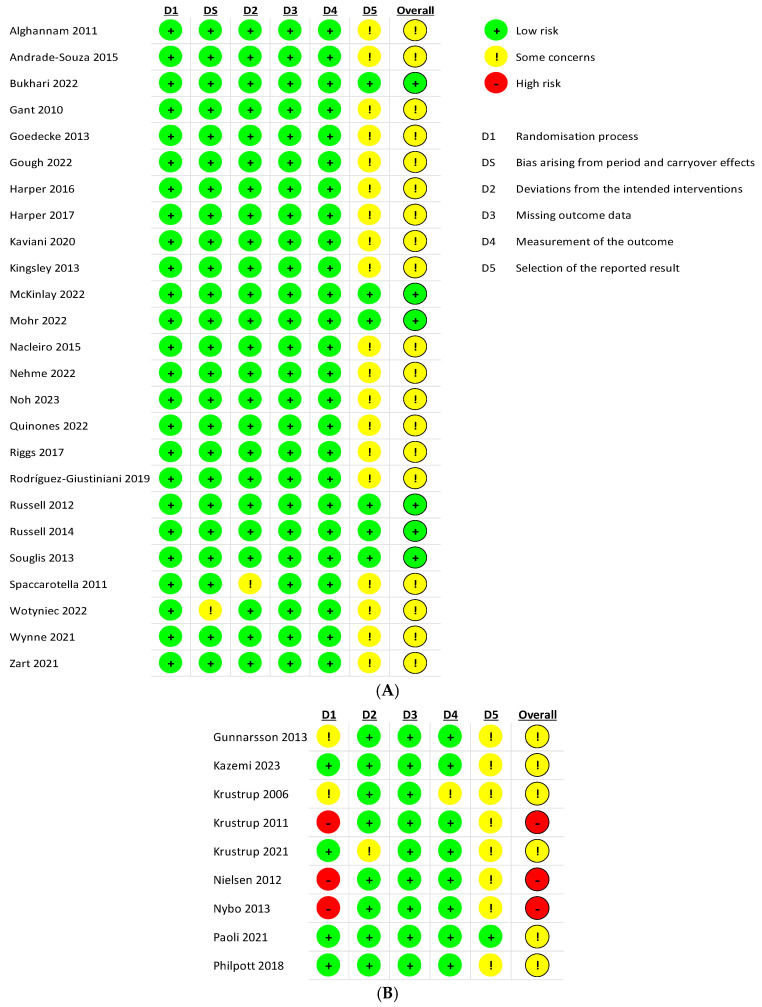
Risk of Bias 2 (RoB 2): crossover studies (**A**) and parallel studies (**B**).

Independently, two authors (M.P. and C.G.) judged the risk of bias of the included studies using Risk of Bias 2 (RoB 2), recommended by the Cochrane Handbook for Systematic Reviews (5.1.0) [31], to assess the risk of bias of the experimental studies included in the review (Figure 2), for both crossover trials (25 studies) [32] (Figure 2A) and parallel trials (9 studies) [33] (Figure 2B). Disagreements were settled by consensus among authors or through consultation with a third reviewer (J.C.).

According to the RoB 2 assessment of the risk of bias for crossover trials, 24% of the studies showed a low risk of bias, 76% showed some concerns about the risk of bias (a moderate risk of bias), and none showed a high risk. Regarding parallel trials, 33.3% of the studies showed a high risk of bias, 66.7% showed some concerns about the risk of bias, and none showed a low risk.

## 3. Results

### 3.1. Physical and Technical Parameters

Table 1 groups 27 clinical trials (24 conducted on men and 3 on women), of which only 4 were on professional soccer players, and 4 descriptive observational studies (2 conducted on men and 2 on women), all involving professional soccer players. The clinical trials include studies that measure the intervention with carbohydrates before, during, and after the match, as well as those that measure it simultaneously (before, during, and/or after the match). Variables are recorded through performance tests and technology and software that allow measuring and collecting the physical performance of the players’ external load. In the descriptive studies, no experimental treatment is applied; performance variables are simply recorded and described through global positioning systems (GPS), which also allow measuring and collecting the players’ external physical load. Some studies measure weekly distance traveled in relation to the number of matches [34,35,36,37,38,39].

#### 3.1.1. Sprints

Men: The ingestion of 30 g of CHO (250 mL at 12%) before each part significantly improves the ability to run with intensity [40], race time, and distance covered [41], as well as sprint speed and number in the last 30 min [42], which is crucial considering that the time spent sprinting 30 m increases in the second half and after intense exercise (*p* < 0.05) [43,44]. Sprint speed was enhanced with a CHO-rich diet before and during the match, supplemented with caffeine (6 mg/kg) [45]. These results were found by Gant et al. [46] also after ingesting 3.7 mg caffeine with 1.8 g/kg CHO vs. 1.8 g/kg of CHO alone before and during a match. However, Briggs et al. [47] did not find sprint improvement after a breakfast containing 77 g of CHO 2 h before, compared to a conventional one with 39 g. Only one study evaluated repeated sprint performance comparing a CHO-based diet and another with CHO and proteins, but no significant differences were found [48]. Consistent with these results, no significant differences were found in sprint performance after completing a demanding soccer test and ingesting CHO, caffeine, or a combination of both [49]. Bukhari et al. [50] demonstrated that supplementation during repeated sprint testing with a 10% dextrose drink (15 g) resulted in higher sprint speeds in soccer players compared to a counterpart with added 20 mM sodium (*p* < 0.05). Gough et al. [51] studied the effect of mouth rinsing with CHO and caffeine (independently or synergistically) on repeated sprint performance after a CHO-rich meal, finding no significant differences.

Kazemi et al. [52] concluded that compared to a control group ingesting 5–6 g/kg/d over 7 days, CHO loading (1.5 g/kg/d on day 1, 1 g/kg/d on days 2 and 3, progressively increasing CHO intake on days 4–7 up to 7.5 g/kg/d in match day [MD]) yielded better results in running (*p* < 0.05), considering total distance covered, maximum speed, and repeated sprint performance. On the other hand, following a diet providing 8 g/kg/d of CHO for 3–4 days allowed soccer players to cover 17% more distance and at higher speeds during the match compared to a diet providing 3 g/kg/d of CHO [53].

Considering only extra time, Harper et al. [54] found no improvement in sprint speed over 15 and 30 m or in sustained 30-m sprinting after consuming electrolyte drinks during the initial 90 min and gels with 0.7 g/kg CHO 5 min before extra time. Data reported during the extra time [55] determined that the total distance per minute decreased by 12% (*p* < 0.05) compared to the initial 90 min of the match (109.7 ± 10.8 m/min vs. 121.5 ± 9.4 m/min, respectively). There was also an 11.6% decrease (*p* < 0.05) in high-speed running per minute (2.05 ± 1.4 m/min at 120 min vs. 2.32 ± 1.35 m/min at 90 min), an 18.3% decrease (*p* < 0.05) in accelerations per minute (0.94 ± 0.3 at 120 min vs. 1.15 ± 0.3 at 90 min), a 16.8% decrease (*p* < 0.05) in decelerations per minute (0.89 ± 0.3 at 120 min vs. 1.07 ± 0.2 at 90 min), a 3.6% decrease (*p* < 0.05) in mean heart rate (162.7 ± 7.7 bpm at 120 min vs. 168.8 ± 8.6 bpm at 90 min), and an 11% decrease (*p* < 0.05) in maximum sprint speed (29.1 ± 2.0 km/h at 120 min vs. 32.4 ± 2.3 km/h at 90 min).

Women: Sprints are less than 10 m and mostly explosive (about 50%), surpassing the number performed by men [37]. Another study measured the effect of consuming a CHO-rich meal (203 g) 4 h before a match vs. a meal rich in mixed macronutrients MM (103 g CHO)—both with the same caloric content—on total distance covered, high-intensity running, and number of sprints, finding no significant differences in any of these parameters [56].

#### 3.1.2. Shooting

Regarding accuracy, success rate, and shot speed, their decline over time was attenuated with the ingestion, in men, of 30 g of CHO at breakfast and 59 g/h during the match (*p* < 0.01) [57].

#### 3.1.3. Passing

There are fewer consensuses regarding this parameter. Rodriguez-Giustiniani et al. [40] found significant improvement in non-dominant foot speed and passing accuracy following pre-match CHO intake and 60 g during the match, whereas other studies in men did not find such improvements [24,46,49,57].

#### 3.1.4. Dribbling

Regarding dribbling, no studies conducted in men [40,54,57,58] found improvements in accuracy or speed except Harper et al. [54], who observed a 29% improvement in accuracy during the extra time after ingesting 0.7 g/kg of CHO (gel) 5 min before its start. On the other hand, two studies found improved dribbling speed after ingesting 30 g of CHO (250 mL at 12%) before each part and after a breakfast containing 77 g of CHO 2 h before, compared to a conventional one with 39 g [42,47].

#### 3.1.5. Jumping and Agility

Men: Data on jumping ability and agility are very limited, with only Kaviani et al. [58] finding improvements in both parameters in the last 20 min after ingesting low GI CHO before and during a match. Goedecke et al. [59] did not find significant results in agility after ingesting 49 g (CHO 7%) during the match. Regarding jumping, only Gant et al. [46] found improvement after consuming 1.8 g/kg CHO combined with 3.7 mg of caffeine. This parameter decreased during extra time despite ingesting 0.7 g/kg of CHO (gel) 5 min before its start [54] and did not improve during the match even when CHO was ingested with caffeine [49].

Women: McKinlay et al. [60] studied the effects of Greek yogurt on recovery after 5 days of intense soccer. When observing jump measurements, no significant differences were found between groups in pre-test and post-test, comparing one group with another, both with the same caloric content (~115 kcal) and less CHO in Greek yogurt (11.5 g GY vs. 28.6 CHO *p* < 0.05). However, this parameter did decrease between pre-test and post-test in both groups when studied separately.

#### 3.1.6. Yo-Yo IR1

Men: Nehme et al. [61] conducted the test after eight single and serial CHO mouth rinses, finding no significant differences in performance. However, performance improved by 12.5% after ingesting 69 g of 7% CHO before and during a rigorous soccer test prior to the Yo-Yo test [62]. Additionally, adding omega-3 fatty acids to CHO-based or CHO + protein diets did not improve Yo-Yo test performance [24]. Nybo et al. [63] found no differences in Yo-Yo IR1 performance 48 h after playing a match at 43 °C compared to one at 21 °C, despite a pre and post-match diet with CHO and proteins.

Women: When observing endurance test measurements, no significant differences were found between groups in the pre-test and post-test, comparing one group with another, both with the same caloric content (~115 kcal) and less CHO in Greek yogurt (11.5 g GY vs. 28.6 CHO *p* < 0.05). However, when studied separately, this parameter decreased between pre-test and post-test in both groups [60].

#### 3.1.7. Ketogenic or Mixed Macronutrient (MM) Diet

Only one study compared a ketogenic diet of <30 g/d CHO (10%) with a Western diet of 50–55% CHO over 30 days [64]. Both diets resulted in weight loss, with reductions in body and visceral fat, extracellular water, and waist circumference in the ketogenic diet, along with similar strength and muscle mass and improved performance (distance covered) in the Yo-Yo IR1 test in both diets. Another study measured the effect of ingesting a meal rich in CHO (203 g) vs. one rich in mixed macronutrients MM (103 g CHO) 4 h before a match—both with the same caloric content—on total distance covered and distance covered at high intensity [56]. No significant differences were found, with the total distance covered being 3.18 ± 0.18 km for MM and 3.24 ± 0.25 km for CHO by the end of the match, of which 416 ± 159 m (MM) and 385 ± 211 m (CHO) were covered at high intensity (≥14.4 km/h) (*p* > 0.05).

### 3.2. Glucose Levels

Table 2 groups 12 clinical trials conducted in men and 1 in women (the only one involving professional soccer players) that study blood glucose levels.

Men: Some studies indicate that glucose levels increased at the end of the first half, during halftime, and in the last 30 min when 30 or 59 g/h of CHO were consumed throughout the match (*p* < 0.05 vs. placebo) [40,42,57], and are higher at the point of fatigue when 0.3 g/kg are consumed along with proteins before each half (*p* < 0.05 vs. placebo and CHO 1 g/kg alone) [65]. However, this increase of up to 30% in glucose levels at the end of the first half was not maintained during halftime and the second half when the CHO solution was 6% [66]. The intake of CHO-enriched drinks along with CHO gels during the match significantly elevated glucose levels at 45 and 90 min [45]. Also, taking 0.7 g/kg of CHO before extra time increased blood glucose by 16% during extra time [54]. Mohr and colleagues [55] had already observed that blood glucose during extra time dropped by 13% compared to the end of the match without exogenous CHO intake. However, when CHO intake was limited to a breakfast intake (77 g or 39 g) 135 min before the match, blood glucose decreased during the match [47]. An important point to highlight is that, despite CHO intake before and during the match, there was a drop in glucose of up to 30% in the first 15 min of the second half [42,45,57,66]. Bukhari et al. [50] evidenced that a drink with 15 g of 10% dextrose showed higher blood glucose concentrations compared to the same sodium drink (+20 mM sodium) after a sprint test (*p* < 0.05). However, the intake of 0.7 g/kg/h of dextrose (8% CHO) before and during halftime of a simulated match did not achieve higher glucose concentrations than those observed with the same amount of low-GI cornstarch supplementation [67].

Women: The study conducted by Krustrup et al. [44], similar to the one conducted on men [43], observed that at the end of the match, glucose levels were increased compared to baseline (*p* < 0.05) after prior intake of a standardized diet.

### 3.3. Muscle Glycogen Levels

Table 3 groups seven clinical trials conducted on men and one on women, of which four were conducted on professional soccer players. These studies investigate muscle glycogen levels through muscle biopsies, generally in the vastus medialis of the thigh.

Men: After a high-level match, glycogen levels drop drastically and remain low for a long time. Nielsen et al. [68] found that the total muscle glycogen volume decreased to 1.7% after the match, rising by 59% the next day and an additional 19% from the second to the fifth day post-match. In the study by Krustrup et al. [43], 50% of the muscle fibers were depleted of glycogen by the end of the match. When an appropriate diet with CHO and proteins was ingested three days before (5 and 1.6 g/kg/day) and after (9.5 and 2.7 g/kg/day), the match, muscle glycogen was 57% lower at the end of the match compared to the control figures of the same subjects (*p* < 0.001), 27% lower at 24 h (*p* < 0.001), while at 48 h there were no significant differences [36]. Additionally, muscle soreness persisted until 72 h post-match versus control (*p* < 0.05) [36]. Similar post-match results were found by Gunnarsson et al. [69], with 50% of muscle fibers depleted of glycogen, even at 48 h vs. baseline (*p* < 0.05) despite a diet with > 8 g/kg/day of CHO, in which a high-CHO diet post-match for 48 h was essential to ensure glycogen resynthesized. Mohr et al. [55] also found that muscle glycogen decreased by 50% in extra time. Nybo et al. [63] found that when examining the muscle after a match played at 43 °C compared to one played at 21 °C, muscle temperature had increased by 1 °C in both groups, and glycogen was decreased in the first 48 h despite a diet with CHO and proteins pre and post-match. The exogenous intake of 30 g CHO-Electrolytes (12%) before each half allowed greater fat oxidation to preserve glycogen stores [41].

Women: Another study conducted by Krustrup et al. [44], similar to the one conducted on men, analyzed muscle glycogen content through biopsies during friendly matches. Glycogen levels decreased by 40% (*p* < 0.05) after intense exercise in the second half and at the end of the match, observing that 69–80% of muscle fibers were depleted or nearly depleted post-match.

### 3.4. Fatigue

Table 4 shows seven clinical trials conducted on men and one on women, and none were conducted on professional soccer players. These studies assess fatigue through perceived exertion scales and performance tests.

Men: The intake of 49 g (7% CHO) during the match increased the time to fatigue with an inversely proportional relationship to body mass (*p* < 0.05) [59]. Alghannam et al. [65] found that the intake of CHO along with proteins, compared to placebo or higher doses of CHO alone, resulted in delayed fatigue and a lower perception of effort (*p* < 0.05) at the beginning and end of the match. Similarly, Nacleiro et al. [48] also observed a lower perception of fatigue compared to placebo (*p* < 0.01) and similar to higher doses of CHO alone. Noh et al. [41] found that the perception of effort was lower with the intake of 30 g of CHO (12%) before each half, but Harper et al. [42] did not observe these differences. Gough et al. [51] studied the effect of CHO and caffeine mouth rinse (independently or synergistically) on a repeated sprints test without finding significant differences in the fatigue index (*p* > 0.05). Mohr et al. [55] measured fatigue-related parameters through the repeated sprint ability (RSA) index and the countermovement jump (CMJ). It was observed that the RSA fatigue index increased by 1.7% at 90 min vs. the start of the match and by up to 6.6% during extra time and that the CMJ decreased by 19% at 90 min vs. the start of the match and by up to 27% during the 120 min.

The clinical trial by Spaccarotella et al. [70] is the only one conducted on both women and men. It compared two CHO drinks, one commercial and one chocolate-flavored with similar energy content, consumed between pre-season training sessions and measured the time running to fatigue. Differences were found in favor of the chocolate drink only when studying men separately, with no significant differences in perceived exertion for both drinks.

### 3.5. Cognition and Gastrointestinal Comfort

Table 5 groups five clinical trials conducted on men and presents data on cognition and gastrointestinal comfort after CHO intake; no trials were conducted on professional soccer players.

The impact on cognitive function and motor skills is improved by CHO intake during the match [57], although sometimes a decrease is observed compared to pre-match levels [42]. Quinones et al. found that supplementation, before each half, with 0.7 g/kg/h of cornstarch (8% CHO) showed a shorter reaction time compared to the same amount of dextrose (*p* < 0.05) but without significant differences in the accuracy rate [67].

A high-calorie breakfast (2079 kcal with 77 g of CHO 135 min before) significantly produced a full stomach and satiety sensation compared to a lighter one without causing worse discomfort [47]. However, the intake of 250 mL of either 12% CHO or electrolytes before each half-increased discomfort in both without significant differences [42]. Regarding the type of simple CHO [71], although subjects reported greater well-being after ingesting sucrose instead of xylitol, these results did not reach statistical significance. However, in terms of tolerance, xylitol was the worst tolerated, causing osmotic diarrhea. Regarding gastrointestinal comfort, sucrose caused more digestive discomfort than fructose and glucose (*p* < 0.05).

### 3.6. Intake and Energy Availability

Table 6 groups 25 observational-descriptive studies (13 conducted on men, 10 on women, and 2 on both men and women), of which 16 were conducted on professional soccer players (>18 years) and 4 on elite youth players (<18 years). These studies report data on energy intake obtained through food record questionnaires completed by the participants of each study, estimating the corresponding energy availability defined as EA: energy availability (energy intake—exercise-induced energy expenditure, kcal/kg).

**In professional male soccer players (>18 years)**: Energy and CHO intake remain within a normal-low range on both match and training days, being higher (*p* < 0.05) on match days (5.1–6.4 g/kg/day and 3.9–4.2 g/kg/day respectively) [35,72,73,74], and during intense vs. moderate training (*p* < 0.05) [74], coinciding with higher energy expenditure on those days [74]. An energy balance is observed at the limit [75], and due to the exogenous intake of gels and drinks [72,73], with suboptimal adherence to recommendations in all MD periods, significantly focusing intake during dinner [76].

During the pre-season period, the intake of 3.6 g/kg/day of CHO does not reach the lower limit recommended by UEFA of 4–8 g/kg/day of CHO for training days, and the average energy availability of the subjects is low (29 kcal/kg/day) [77].

As a recovery strategy, 90% of the players use CHO, mainly on match days, with a significant relationship (*p* < 0.01) regarding their perception of its effectiveness, especially in post-match consumption [78].

**In male youth soccer players (<18 years)**, including those who are already professionals, the younger they are, the stricter they are in following the weekly CHO intake recommendations (6 ± 1 g/kg/day), with significant differences compared to adults, and with a similar pattern, focusing CHO intake during dinner. Despite this, none meet the energy and CHO intake recommendations both before and after training and matches [75,79,80,81,82,83] and show an unbalanced distribution throughout the day [75,80].

Women: The high percentage of soccer players with low EA (<30 kcal/kg/day) during the pre-season and the season [39,84,85], 26.3% and 33.3% (*p* < 0.05 vs. 11.8% post-season) [86], and even reaching up to 67% [87], is noteworthy. Around 62% of the players show reduced EA (30–45 kcal/kg/day) during the season [84,85]. Moreover, EA tends to be higher on rest days compared to training and match days and during light training compared to intense training [85]. These figures correlate with a low energy intake (*p* < 0.05) below the recommended levels during the pre-season [38,39,84,85,86,87,88,89], especially during post-exercise recovery phases [90], mainly due to low CHO consumption (3.2–5 g/kg/day) [38,39,84,85,86,87,88,89,91], which tends to be higher the day before and on match day [84], higher in matches than in training sessions [39], and also during periods when exercise alone can exceed 800 kcal [86]. Players prefer low-CHO (2%) CHO-electrolyte drinks (compared to 6% CHO drinks) instead of food (bananas or gels) [91].

These energy requirements on training and match days for both men and women are comparable and do not meet the recommendations [38]. EA tends to remain at optimal levels (>45 kcal/kg/day) only on rest days and light training days [85], varying according to sample size and manifesting in 38% of soccer players in the study by McHaffie et al. [84] and 15% in Moss et al. [85].

Only two descriptive studies have compared CHO intake in men and women [92,93]. In one study by Sebastiá-Rico et al. [92], CHO intake was found to be lower in women, while in another study by Gomez-Hixson et al. [93], no significant differences were found between both sexes. However, in both studies, CHO intake levels were below the recommended levels.

## 4. Discussion

The purpose of this study is to analyze the influence of CHO intake on the physical and technical aspects, glucose and muscle glycogen levels, fatigue, cognition, and gastrointestinal comfort involved in soccer players’ performance. It also aims to understand the current availability of energy and investigate whether there are differences between men and women to guide future research and improve nutrition strategies in soccer.

It is worth noting that most of the studies included in this review are conducted in men, and the few studies conducted in women are primarily descriptive observational, mainly focused on energy availability. This may be due to the higher number of licenses in the male field that facilitate their access and the greater scope that economic interests derived from their results may have. Additionally, there are few studies on professional soccer players included in this study, as it is possibly difficult for them to consent both individually and for the clubs to intervene in their training and diets, given everything at stake regarding results and financial status.

Soccer players are becoming increasingly athletic, requiring high performance throughout the match [94]. In male and female soccer, the total distance covered, and the distance covered at high intensity are similar and greater during matches compared to training days, which decreases as the competition day approaches. Diets high in CHO in the days before the match are associated with a greater distance covered (17%) at any speed than with low diets [53] and reaching higher heart rates, with greater efficiency and less fatigue. This may be because they arrive at the match with full glycogen stores to meet the game’s demands.

It is noteworthy in women’s soccer that sprints are shorter but more explosive and in greater numbers than in men [37], which may indicate a different style of play where explosive and short-distance movements are common. This suggests that training for sprints of greater distance than are often required during the match would be necessary. The intake of CHO (30 g) before each half significantly improves the ability to run with intensity, distance covered, speed, and number of sprints in the last 30 min [40,41,42,43]. Sprinting and distance covered also improve with the addition of caffeine to CHO [45], as an ergogenic aid capable of stimulating the central nervous system and enhancing its oxidation when co-ingested with CHO.

Russell et al. [57] added that the decrease in accuracy, success rate, and shooting speed was mitigated by the intake of 30 g of CHO in breakfast and 59 g/h during the match. There are no conclusive data regarding the effect on passing, dribbling, jumping, and agility. Rodriguez et al. [40] observed that the intake before and during the match of CHO leads to making more passes with both legs without loss of speed and even with better accuracy with the non-dominant leg. The impact of CHO on dribbling is not as clear. However, Harper et al. [54] found higher accuracy in extra time after the intake of gels before its start, as well as Wynne et al. [56] in the execution speed, possibly associated with an increase in brain glucose that preserves the integrity of the central nervous system in moments of fatigue. Regarding jumping and agility, only two studies found improvement in the last 20 min [46,58]. In relation to these parameters, mouth rinses have not been shown to have effects on intermittent team sports like soccer [51,61], where the response to these is individual for each athlete, so more studies would be needed.

In soccer, an adequate intake of CHO is essential to maintain glycogen stores in the liver, blood glucose concentration, and late-match performance [94]. During exercise, hepatic glucose is released into the blood, some of which nourishes the brain while the rest supports muscle stores, necessitating sufficient levels throughout the match [54]. Hence, elevated glucose concentrations, as a means to monitor CHO availability in the body, are associated with improved skills compared to euglycemia [40,42]. In this context, the increase in adrenaline following high-intensity actions also stimulates glycogenolysis. It increases glucose production in the liver while directly inhibiting insulin release, leading to elevated glucose levels [40,42,57,66]. These levels remain low at the beginning of the second half. This transient hypoglycemia seems independent of the amount of CHO ingested and tends to decline when high glycemic index CHOs are consumed initially [16]. However, although in sports in general, it is attributed to increased insulin during passive periods after CHO intake, in soccer, it minimally affects performance. It is hypothesized that despite glucose absorption by previously active muscles, the increase in catecholamines and cortisol during the match mitigates the insulin spike [10].

To maintain performance throughout the match, preserving hepatic and muscular glycogen stores is necessary, especially considering that matches now extend beyond 90 min, even into penalties, which reduces physical performance and increases the likelihood of injuries over time [11,94]. Hepatic glycogen recovers more quickly but also depletes faster. Up to 33% can be lost after an overnight fast and up to 50% before the start of the match if CHOs are not consumed [6]. Additionally, given the varying schedules dictated by television rights, attention must be paid to the CHOs consumed at breakfast and the main meal [95]. It is essential to consider the quantity and quality of CHOs in the diet throughout the week. Therefore, preference should be given to complex CHOs like starches, which mitigate increases in blood insulin levels, thereby maintaining fat mobilization and oxidation during exercise and avoiding the long-term consumption of large amounts of simple sugars. Thus, athletes should be aware of these aspects and adopt a diet, choosing the appropriate type of CHO for each moment [53]. Scientific literature shows strong correlations between the muscle’s beta-oxidative capacity and the ability to maintain performance towards the end of a match following CHO intake, indicating a performance-enhancing effect due to increased fat oxidation, likely due to glycogen preservation [9,15,41]. Post-match recovery also depends on CHO intake, as studies demonstrate deficient glycogen levels in muscle fibers at the end of a soccer match in both sexes [6,36,43,44,68]. The incomplete restoration of initial glycogen levels could be linked to muscle damage resulting from repeated accelerations and decelerations, which hinders synthesis [8]. It typically takes 48–72 h to completely replenish muscle glycogen stores, particularly in type 2 fibers [6,69]. In this race against time, given the current accumulation of matches, nutritional strategies beyond cold-hot water immersion, massages, and recovery exercises are needed. A CHO-rich post-match diet for 48 h is essential to ensure glycogen resynthesized and recovery [34,96], although it will depend on the minutes played. Nybo et al. [63] found that when examining muscle after a match at 43 °C compared to 21 °C, in both groups, glycogen was depleted in the first 48 h despite a CHO and protein diet pre- and post-match, as heat increases glycogen use, but running is less intense and shorter [73]. Additionally, at normal temperature, the muscle also heats up and gets damaged.

Performance is affected by both motor and perception fatigue. Mental fatigue creates a feeling of tiredness, impacting the physical execution of technical and tactical decisions, including the most essential skills [9]. It often fluctuates both during a match and throughout the season [6]. It stands to reason that skills will also be better executed if CHO intake improves and delays the onset of fatigue. Therefore, it has been observed that the time to fatigue increased significantly with the intake of 49 g (7% CHO) during the match [59] and resulted in a lower perception of effort when ingested with proteins [48,65]. Sports performance depends on motor skills, cognitive state, perception, and interaction with the environment [8,16], all of which are affected by fatigue, leading to an increase in the number of goals scored towards the end of the match. Interesting studies by Zhu et al. [97,98] show that applying mindfulness along with CHO during halftime improves recovery from mental fatigue and cognitive ability and reduces stress, anxiety, exhaustion, and pain compared to CHO alone or placebo (*p* = 0.02). However, it is a technique that requires extensive prior training.

At times, the lack of CHO intake during matches is due to a fear of poor gastric tolerance and a feeling of fullness. However, this fear can be effectively managed by combining different CHOs to improve their absorption. In this sense, CHOs—more specifically, monosaccharides with a high glycemic index (GI)—absorb faster and are more digestible, accelerating the supply of glucose to the systemic circulation and thus to the skeletal muscles [8,9,17]. Therefore, they are also indicated when the goal is to restore glycogen as quickly as possible, although their influence on performance, such as sprinting or endurance, is unclear compared to low GI CHOs [7,20,29]. Additionally, the intake of low volumes of CHO is associated with low gastrointestinal discomfort [99].

Another important aspect to consider is energy availability (EA), where there is generally insufficient energy intake (EI). It has been shown that insufficient EI causes loss of body and muscle mass, injuries, illnesses, increased prevalence of overtraining, and severe performance deterioration [3,8] across all age groups and categories [17]. There has been a trend in the last 14 years of low energy intake relative to energy expenditure, starting from early ages, especially on competition days, which does not occur with other macronutrients [100]. Young players experience rapid growth and maturation changes that demand higher nutritional needs [17]. However, this can be compromised as they barely meet the lower limit of requirements, which means they may start matches with depleted glycogen stores and reduced fuel availability. This is especially important in women [92,93], posing a health risk. Notably, Reed et al. found an inverse relationship between EA and body dissatisfaction and the desire to be thinner [86], highlighting the fear of CHO intake in elite female soccer players [84]. Scientific evidence shows considerable confusion and misconceptions among female players regarding current nutritional guidelines for soccer, often due to preconceived notions about the perceived impact of CHOs on body composition, something that only occurs when large amounts are consumed over a long period without exercise. This is compounded by external pressures from coaches, support staff, parents, social media, and the culture surrounding body composition measurements. The lack of experience regarding specific female health among staff members can be especially problematic in cases where players exhibit symptoms related to the female athlete triad [15]. Therefore, it is crucial for sports nutritionists and support staff to guide athletes in maintaining adequate CHO intake and adjust it according to the intensity and energy demands of the work [8,10,101].

The findings support the need to educate players, parents, coaches, and physical trainers about the importance of proper nutrition for physical and mental health, as well as for improving performance [3,8,15,91]. As a final consideration, it is necessary to periodize CHO intake according to each training session and match, not forgetting recovery and considering the individualization of each player based on their characteristics, position on the field, and playing time [22,101].

In the future, more research is needed on women to determine if female soccer players would benefit from different dietary recommendations than men to optimize their physical training results. Studies should investigate the impact of the menstrual cycle on nutritional habits, performance, and recovery; nutritional needs according to position on the field, as there is significant inter- and intra-individual variability in work rate and physiological load during matches; and nutritional strategies for vegan and vegetarian players, who are becoming increasingly numerous.

## 5. Limitations

One of the limitations of this study is that in soccer, research on CHO intake is mainly based on laboratory models or simulations, whose results may fail to replicate on match day. Many studies are conducted on amateur players and not on professionals; the methodology is very varied and flawed, with few cases; there are few studies on female soccer players or both sexes or that include juniors or referees; and there are biases, especially in supplements and specific diets, where negative results are unlikely to be published, requiring more significant evidence and consensus. Additionally, studies are needed to adapt nutritional strategies to the position and activity of each player during the match and to study factors that improve the rate of glycogen synthesis and storage in a limited time from a limited intake [17,27,94].

## 6. Conclusions

CHOs are the primary source of energy in soccer, and muscle and liver glycogen depletion are a decisive factor for performance. CHO intake before and during the match is associated with improved speed and number of sprints, attenuates the decrease in shooting accuracy and speed throughout the match, increases the time to fatigue, and improves cognitive function.

Consuming 6–8 g/kg/day of CHO the day before is recommended, as a meal with 1–3 g/kg 3–4 h before and 30–60 g/h during the match. Muscle glycogen levels drop drastically at the end of the match and remain low for 48 h, so consuming 1–1.5 g/kg/h for 4 h is recommended, starting within the first 20 min. On training days, in weeks with one match, 3–6 g/kg/day is recommended, increasing to 6–8 g/kg/day before and after the match. During periods of match congestion, the recommended amount is 6–8 g/kg/day daily.

For female soccer players, who face similar physical demands as men, it has been observed that energy availability is low, especially in the post-match periods, as they tend to underestimate energy expenditure and consume fewer CHO. It is crucial for soccer players to fully comprehend and adhere to dietary recommendations for training and competing. This is essential to maintain high glycogen stores, keep blood glucose levels stable, and ensure optimal physical, technical, and cognitive performance.

## Figures and Tables

**Table 1:** Physical and technical aspects.

Authors	Sample	Intervention	Variables	Effects
Philpott et al. (2018) [24] CT	*n* = 30 Male competitors 23 ± 1 years UK	**MORNING AND EVENING—6 WEEKS (wk)** 1. **FO** (omega3): 1100 mg omega-3 fatty acids + 15 g protein + 1.8 g leucine + 20 g CHO 2. **PRO** (protein): 15 g protein + 1.8 g leucine + 20 g CHO 3. CHO: 24 g → 2 × 200 mL (1 morning and 1 evening; 6 wk)	Passing ability test (s): **FO** 35 s vs. **PRO** 37 s **CHO** 42 s Yo-Yo IR2 (m): **FO** 490 m vs. **PRO/CHO** 480 m	**↔** **↔**
Anderson et al. (2016) [34] ODS	*n* = 12 Professional men 25 ± 5 years UK	1 week: 1 MD + 4 TD 1 week: 2 MD + 4 TD 1 week: 3 MD + 2 TD	**1 MD + 4 TD:** Daily distance covered (m): **MD-3** 5223 ± 406; **MD-2** 3097 ± 149; **MD-1** 2919 ± 192 Weekly distance (km): 25.1 ± 2 **2 MD + 4 TD:** Daily distance covered (m): similar to **1 MD + 4 TD** Weekly distance (km): 32.5 ± 4.1 **3 MD + 2 TD:** Daily distance covered (m): **MD-1**: 2422 ± 251 Weekly distance (km): 35.5 ± 2.4 Time spent > 14 km/h	**↑MD-3** *p* < 0.05 vs. **MD-2** and **MD-1** *p* < 0.05 vs. **1 MD** + **4 TD** *p* < 0.05 vs. **1 MD** + **4 TD** and **2 MD** + **4 TD** *p* < 0.05 vs. **1 MD** + **4 TD** *p* < 0.05 vs. **1 MD** + **4 TD** and **2 MD** + **4 TD**
Brinkmans et al. (2019) [35] ODS	*n* = 41 Professional men 23 ± 4 years Netherlands	14 days	**MD and TD Load:** Duration (min): **MD** 90 ± 6 vs. **TD** 78 ± 11 Total distance covered (m): **MD** 10,293 ± 1710 vs. **TD** 5405 ± 835 Distance covered per minute (m/min): **MD** 115 ± 19 vs. **TD** 70 ± 8	**↑ MD** *p* < 0.001 (all measurements)
Krustrup et al. (2011) [36] CT	*n* = 7 Professional men 27 ± 1 years Denmark	**BEFORE, AFTER** **Diet**: **CHO** (g/kg/d): **CHO** (9.5), **Proteins** (2.7), and **Fats** (0.8) (67%-20%-13% energy) + creatine. Post-match: for 5 days. → muscle biopsy and blood test → 3 days before the match: CHO 5 g/kg/d (55%), 1.6 Prot	**During match: 11.31 km, 1.68 km high intensity** **Glycogen 0 h** **Glycogen 24 h** **Glycogen 48 h** **Muscle soreness at 0, 24, 48, and 72 h**	**↓** 57%, *p* < 0.001 vs. control **↓** 27%, *p* < 0.001 vs. control No differences, *p* = 0.096 **↑** *p* < 0.05 vs. control
Datson et al. (2017) [37] ODS	*n* = 107 Professional women UK	Demand in an international match	**Total distance covered (m):** - Central Midfielders (CM): 10,985 ± 706 - Central Defenders (CD): 9489 ± 562 **Total distance covered at high speed (m) (>14.4 km/h):** - Central Midfielders (CM): 2882 ± 500 - Central Defenders (CD): 1901 ± 268 **Total distance covered at very high speed (m) (>19.8 km/h):** - Without ball: (399 ± 143) - With ball: (313 ± 210) **Short-distance sprints:** 76% under <5 m; 95% under <10 m **Explosive sprints (%) women:** 51 ± 10	**↑ CM** (similar to men) ** ↑ CM** (similar to men) Greater than in men
Morehen et al. (2022) [38] ODS	*n* = 24 Professional women UK	12 days international camp: 4 TD, 1 MD, rest, travel, 1 TD, 1 MD, travel, 3 rest. **→** 13 soccer players used GPS	**Training duration (min):** - MD-4: 89 ± 4 - MD-3: 89 ± 5 - MD-1 1st match: 61 ± 2 - MD-1 2nd match: 63 ± 7 - MD 1st match: 64 ± 33 - MD 2nd match: 73 ± 31 **Total distance covered (m):** - MD-4: 6020 ± 620 - MD-3: 6340 ± 53 - MD-1 1st match: 2927 ± 862 - MD-1 2nd match: 4063 ± 540 - MD 1st match: 6243 ± 340 **Total distance covered at high speed (m):** - MD-4: 126 ± 85 - MD-1 1st match: 85 ± 79 - MD-1 2nd match: 77 ± 41 - MD 1st match: 361 ± 183 - MD 2nd match: 337 ± 197	**↑ MD-4** vs. **MD-1 1st** and **2nd match** *p* < 0.05 **↑ MD-3** vs. **MD-1 1st** and **2nd match** *p* < 0.05 **↔ MD 1st** and **2nd match** vs. **rest of days** *p* > 0.05 **↑ MD-4** vs. **MD-1 1st** and **2nd match** *p* < 0.05 **↑ MD-3** vs. **MD-1 1st** and **2nd match** *p* < 0.05 **↓ MD-1 1st match** vs. **MD-1 2nd match** *p* < 0.05 **↔ MD 1st match** vs. **rest of days** *p* > 0.05 **↑ MD 1st match** vs. **MD-4, MD-1 1st** and **2nd match** *p* < 0.05 **↑ MD 2nd match** vs. **MD-1 1st** and **2nd match** *p* < 0.05
Rodriguez-Giustiniani et al. (2019) [40] CT	*n* = 18 Academy Elite Youth Men 18 ± 2 years old UK	**BEFORE, DURING** **1. CHO (12%) + electrolytes:** 250 mL before each half of the simulated match, 60 g total CHO **2. Placebo** → All, breakfast 46 g CHO and 2 g/kg at lunch 2 h before the match	**Speed pass dominant foot** **Speed pass non-dominant foot** **Passing Accuracy** **High intensity running capability** **Dribbling speed/accuracy**	**↔** **↑ CHO**, *p* < 0.05 **↑ CHO**, *p* < 0.05 **↑ CHO**, *p* < 0.05 **↔**
Noh et al. (2023) [41] CT	*n* = 8 College Men 21.32 ± 1.19 years South Korea	**BEFORE, DURING** **1. CHO-E:** 60 g (2 × 30 g): (20 g at 12%, <5 mg sodium (Na) and <0.5 g prot) (500 mL) **→** 15′ before exercise and at rest (mixed) **2. Placebo-E** (500 mL) **→** 15′ before exercise	**Running Time (s): Placebo** 422.13 ± 133.44 vs. **CHO-E** 677.38 ± 217.7 **Distance (m): Placebo** 1577.25 ± 517.02 vs. **CHO-E** 2530.00 ± 832.71	**↑ CHO-E vs. Placebo** *p* < 0.05 **↑ CHO-E vs. Placebo** *p* < 0.05
Harper et al. (2017) [42] CT	*n* = 15 College Men 22 ± 2 years UK	**BEFORE, DURING** **1. CHO Drink (12%) + Electrolytes:** 60 g Total CHO **2. Placebo (electrolytes)** **3. Water** **→** 15′ before each half of the simulated match (250 mL). All, breakfast with 35 g CHO, 135′ before	**Sprint 15 m (m/s): CHO** 5.4 vs. **Placebo** 5.23 vs. **Water** 5.2 **No. self-paced accelerations: CHO** 9 ± 4 vs. **Placebo** 7 ± 4 vs. **Water** 6 ± 4 **Dribbling speed (m/s): CHO** 5.4 vs. **Placebo** 1.15 vs. **Water** 1.15	**↑ CHO,** *p* < 0.05 **↑ CHO,** *p* < 0.05 vs. placebo and water, last 30′ **↑ CHO,** *p* < 0.05 vs. placebo and water, last 30′
Krustrup et al. (2006) [43] CT	*n* = 31 men 21–33 years Denmark	**BEFORE, DURING, AFTER** All standardized diet.	**Time to do the sprint**	**↑** *p* < 0.05 in the 2nd half, post and after intense exercise
Krustrup et al. (2022) [44] CT	*n* = 20 Professional women 23 ± 4 years Denmark	After 5 sprints of 30 m with a rest of 25 s. All standardized diet.	**Time to do the sprint**	**↑** *p* < 0.05 at the end of both halfs and after intense exercise
Kingsley et al. (2014) [45] CT	*n* = 14 Amateur men 24 ± 1 years UK	**BEFORE, DURING** **1. H-CHO Drink:** 9.6% CHO + Caffeine (6 mg/kg) + Electrolytes/+ Active Gels (CHO 37.3 g + Electrolytes) **2. CHO Drink**: 5.6% CHO + electrolytes **3. Placebo** **→** 500 mL at breakfast; before each half 5.25 mL/kg and 2 gels (60 g) and 2.6 mL/kg every 15′/simulated match	**Average sprint speed (m/s):** **H-CHO** 5.73 ± 0.08 vs. **CHO:** 5.66 ± 0.08 vs. **Placebo** 5.58 ± 0.10	**↑ H-CHO,** *p* < 0.05 vs. placebo
Gant et al. (2010) [46] CT	*n* = 15 Men’s Regional Premier League 21.3 ± 3 years	**BEFORE, DURING** **1. CHO**: 1.8 g/kg CHO **2. CAF (caffeine):** 1.8 g/kg CHO + 3.7 mg/kg caffeine **→** 1 h before and every 15’ of exercise in a simulated match	**Jump (cm): CAF** 52.9 ± 5.8 vs. **CHO** 51.7 ± 5.7 **Average time in sprints 15 m (s): CAF** 2.48 vs. **CHO** 2.59 at end **Passing Ability**	**↑ CAF,** *p* = 0.03 **↓ CAF,** *p* = 0.04 **↔**
Briggs et al. (2017) [47] CT	*n* = 7 Men’s Youth Premier Academy 16 ± 1 years UK	**BEFORE** **1. Rich Calorie Breakfast (RCB):** 2079 kJ, 77 g CHO, 14 g Prot and 12 g Fat **2. Usual breakfast (UB):** 1122 kJ, 39 g CHO, 10 g Prot, and 8 g Fat **→**135′ before the match, on 2 different days	**Jump and sprint speed** **Average dribbling speed**	**↔** **↑ in RCB**, *p* = 0.023
Naclerio et al. (2015) [48] CT	*n* = 16 Amateur Men 24 ± 3.7 years UK	**BEFORE, DURING, AFTER** **1. MTN** (53 g CHO + 14.5 g Prot + 5 g Glutamine + 1.5 g L-carnitine-L-tartrate) × 2 (total 106 g CHO) **2. CHO** 69.5 g × 2 (total 139 g) **3. Placebo** **→** before-during repeated sprint test in 90′ (4 blocks separated by 3′ rest) and 20′ after	**Repeated sprint performance**	**↔**
Andrade-Souza et al. (2015) [49] CT	*n* = 11 Amateur Men 25.4 ± 2.3 years Brazil	**AFTER** **1. CHO:** 1.2 g/kg/h CHO **→** immediately after and 1, 2 and 3 h post LIST **2. CAF** (caffeine): 6 mg/kg **→** 3 h post LIST **3. CHO + CAF** **4. Placebo**	**Performance after 4 h post LIST: jumps, passes, sprints**	**↔** in all groups
Bukhari et al. (2022) [50] CT	*n* = 22 Young men 19.6 ± 1.1 years Indonesia	**DURING** **1. Dextrose (DEX):** 15 g in 150 mL (10%) **2. Sodium Dextrose (Na-D):** 15 g in 150 mL (10%) + 20 mM sodium (Na) **→** 2 × 100 m, and sprint speed. Rest 15′, DEX or Na-D drink, rest 15′, run 2 × 100 m and sprint o Na-D, rest 15′, run 2 × 100 m and sprint	**Average sprint speed (s):** - Before: **DEX** 14.6 ± 1.33 vs. **Na-D** 15.2 ± 1.25 - After: **DEX** 14.6 ± 1.46 vs. **Na-D** 15.9 ± 1.61	**↑ DEX** vs. **Na-D** *p* < 0.05
Gough et al. (2022) [51] CT	*n* = 9 Amateur men 21 ± 3 years UK	**DURING** **1. CHO +CAF:** 10% maltodextrin + 6 mg/kg/1 caffeine **2. CAF (caffeine):** 6 mg/kg/1 **3. CHO:** 10% maltodextrin **4. Placebo**: water **→** 4 × 90′ Soccer-Specific Aerobic Field Test (SAFT90) and two series of repeated sprint ability tests (RSA; 6 × 6 s sprints with 24 s recovery) completed at 0′ and 75′ of SAFT90 → **mouthwash:** 25 mL immediately before the 2nd RSA (minute 75)	**Mean Power Output (MPO) (W)**: ** 1st RSA 0′** 213 ± 29 vs. **RSA 75′** 215 ± 31 ** 2nd RSA 0′** 221 ± 24 vs. **RSA 75′** 226 ± 27 ** 3rd RSA 0′** 226 ± 21 vs. **RSA 75′** 223 ± 29 ** 4th RSA 0′** 225 ± 22 vs. **RSA 75′** 225 ± 24 ** Peak Power Output (PPO) (W**): ** 1st RSA 0′** 229 ± 29 vs. **RSA 75′** 233 ± 33 ** 2nd RSA 0′** 234 ± 27 vs. **RSA 75′** 226 ± 27 ** 3rd RSA 0′** 226 ± 21 vs. **RSA 75′** 223 ± 29 ** 4th RSA 0′** 239 ± 24 vs. **RSA 75′** 243 ± 29	**No differences** *p* > 0.05
Kazemi et al. (2023) [52] CT	*n* = 22 Professional men CHO: 28.4 ± 3.0 years CON: 29.2 ± 4.19 years Iran	**1. CHO load: ↓ CHO** 3 d (1.5 day 1, 1 g/kg/d days 2 and 3) and increased exercise intensity. 4–7 days, **↓** exercise intensity and **↑** CHO increased up to 7.5 g/kg/d in MD **2. CONTROL (CON):** same physical training but **↔** CHO of 5–6 g/kg/d	**Running distance (m): CHO** 12.64 ± 1.39 vs. **CON** 8.22 ± 1.13 **Top speed (km/h): CHO** 32.9 ± 4.97 vs. **CON** 29.4 ± 5.77 **Repeated Sprints (RSA) performance (m/s): CHO** 81 ± 19.57 vs. **CON** 60.81 ± 20.18	**↑ CHO** vs. **CON** *p* < 0.05 **↑ CHO** vs. **CON** *p* < 0.05 **↑ CHO** vs. **CON** *p* < 0.05
Souglis et al. (2013) [53] CT	*n* = 22 Professional men HC: 24 ± 0.7 years LC: 24.5 ± 0.8 years Greece	**1. HC (high CHO): 8 g/kg/d** **2. LC (low CHO): 3 g/kg/d** → 3–5 days before the match at the end of the season	**Total distance covered (m): HC** 9.380 ± 98 vs. **LC** 8.077 ± 109	**↑ 17% HC and at high speeds** *p* < 0.05
Harper et al. (2016) [54] CT	*n* = 8 Young men Premier Academy 16 ± 1 years UK	**BEFORE, DURING, AFTER** Analyze 120′ (90′+ overtime), simulated × 2 **→** All drinks with electrolytes (E) before, in the middle and at 90′, 5′ before extra time gel: **placebo** or 0.7 g/kg **CHO/E**	**Sprint speed 15 and 30 m, Sprint maintained 30 m, Jump, Dribble speed ** **Precision dribbling**	**↓** in extra time in both groups **↑** in extra time with CHO/E (+29%), *p* < 0.01
Mohr et al. (2023) [55] CT	*n* = 20 Men competition 20.1 ± 1.4 years	**→** 2 matches of 120’ separated by 3 days **→** At the beginning and after 90 and 120’ of the match **→** GPS	**Total distance (m/min): Normal time** 121.5 ± 9.4 vs. **ET** 109.7 ± 10.8 **High intensity running (m/min): Normal time** 2.32 ± 1.35 vs. **ET** 2.05 ± 1.4 **Accelerations/min (>2 m·s ^−2^): Normal time** 1.15 ± 0.3 vs. **ET** 0.94 ± 0.3 **Decelerations/min (>2 m·s ^−2^): Normal time** 1.07 ± 0.2 vs. **ET** 0.89 ± 0.3 **Maximum sprint speed (km/h): Normal time** 32.4 ± 2.3 vs. **ET** 29.1 ± 2.0	**↓ 12%** in **ET** vs. **normal time** *p* < 0.05 **↓ 11.6%** in **ET** vs. **normal time** *p* < 0.05 **↓ 18.3%** in **ET** vs. **normal time** *p* < 0.05 **↓ 16.8%** in **ET** vs. **normal time** *p* < 0.05 **↓ 11%** in **ET** vs. **normal time** *p* < 0.05
Wynne et al. (2021) [56] CT	*n* = 15 Women University League 19.6 ± 1.3 years USA	**1. MM: mixed macronutrients** ~1000 kcal, 1.5 g/kg (103 g) CHO **2. HCHO: ↑ CHO**, ~1000 kcal, 3–4 g/kg (203 g). → 4 h before the 70′ match	**Total distance traveled (km):** 1st half: **MM** 3.43 ± 0.22 vs. **HCHO** 3.44 ± 0.30 2nd half: **MM** 3.18 ± 0.18 vs. **HCHO** 3.24 ± 0.25 **High intensity running (m) HSR; ≥14.4 km/h:** 1st half: **MM** 416 ± 159 vs. **HCHO** 433 ± 204 2nd half: **MM** 330 ± 141 vs. **HCHO** 385 ± 211 **Sprint count (no.) > 17.1 km/h, maintained for at least 1 s:** 1st half: **MM** 10.1 ± 5.5 vs. **HCHO** 11.3 ± 6.9 2nd half: **MM** 9.1 ± 6.0 vs. **HCHO** 10.8 ± 6.7	**No differences** *p* > 0.05 **No differences** *p* > 0.05 **No differences** *p* > 0.05
Russell et al. (2012) [57] CT	*n* = 15 Young men Academy 18 ± 1 years UK	**BEFORE, DURING** **1. CHO (6%) + electrolytes** **2. Placebo (electrolytes)** **→** 500 mL breakfast + 3.5 mL/kg 10′ before each half and after 15, 30, 60, and 75′ of exercise (59 g CHO/h), simulated match	**Accuracy, success rate, and shoting speed** **Accuracy and passing speed** **Accuracy, success rate, and dribbling speed**	CHO attenuates its ↓ in time, *p* < 0.05 **↔** **↔**
Kaviani et al. (2020) [58] CT	*n* = 8 Amateur men 30 ± 7 years Canada	**BEFORE, DURING** **1. CHO High Glycemic Index (GI)** **2. CHO Low GI** **→** Simulated match; 1.5 g/kg 2 h before + 0.38 g/kg at rest	**3′ dribbling intervals, agility, running, jumping.**	**↑** agility and jump at 72′ low GI vs. high GI (*p* < 0.01)
Goedecke et al. (2013) [59] CT	*n* = 22 Amateur men 24 ± 7 years Southafrica	**DURING** **1. CHO (7%)** → 49 g (28 g/h if heating is included) **2. Placebo** → 700 mL during simulated match (× 2, 7 days apart)	**Agility**	**↔**
McKinlay et al. (2022) [60] CT	*n* = 13 Female Cadets 14.3 ± 1.3 years Canada	**1. GY (Greek yogurt): 3 servings/day of 160 g** (~115 kcal, 17 g protein, ~11.5 g CHO) **2. CHO: 30 g** (CHO, ~115 kcal, 0.04 g protein, ~28.6 g CHO) **→** for two 5-day soccer-specific training camps, after training, 1 h before bedtime, between breakfast and lunch the next day	**CMJ (cm):** - PRE: **GY** 22.6 ± 1.0 vs. **CHO** 22.8 ± 1.0 - POST: **GY** 22.0 ± 1.0 vs. **CHO** 21.9 ± 1.1 **Beep test distance (m):** - PRE: **GY** 1270.7 ± 70.5 vs. **CHO** 1303.1 ± 72.0 - POST: **GY** 1136.9 ± 50.1 vs. **CHO** 1170.7 ± 47.8	**↔ pre** vs. **post between both groups** *p* > 0.05 **↓ post** vs. **pre in both groups** *p* < 0.05 **No differences pre between groups** *p* > 0.05 **↓ post** vs. **pre in both groups** *p* < 0.05
Nehme et al. (2022) [61] CT	*n* = 12 Amateur men 18–20 years Brazil	**BEFORE** **8 mouthwashes:** **1. Placebo:** 8 with placebo **2. Placebo + CHO:** 7 with placebo + 1 with CHO (8% maltodextrin) **3. 8-CHO:** 8 CHO Rinses (8% Maltodextrin) **→** Pre-test Yo-Yo IR1 to assess maximal aerobic endurance performance measured through total distance traveled	**Yo-Yo IR1 (m): Placebo + CHO** (1.198 ± 289 m) vs. **8-CHO** (1.256 ± 253 m) vs. **placebo** (1.086 ± 284 m)	**No differences between groups** *p* > 0.05
Zart et al. (2021) [62] CT	*n* = 9 Amateur men 23.3 ± 2.4 years	**BEFORE, DURING** **1. CHO Syrup**: 69 g (7%) **2. Placebo:** aromatic water **→** 3 days of trial, separated by 1 week. 6 × 4′ Hoff tests with 3′ active rest between each load and then Yo-Yo IR1 until exhaustion **→ Day 2 and 3:** 250 mL 30′ before, immediately before and during exercise	**Total distance covered (m): Placebo** 826.67 ± 260.77 vs. **CHO** 920.00 ± 256.12	**↑ CHO** vs. **Placebo** *p* < 0.05 **(↑ 12.5% performance)**
Nybo et al. (2013) [63] CT	*n* = 17 Semi-professional men 27 ± 1 years Denmark	**BEFORE, AFTER** Samples muscle and blood before, and 0, 24, 48 h post-match (43 °C vs. control at 21 °C) **→** Diet before and 48 h post-match (energy): 60% CHO, 15–20 Prot and 20–25 Fats	**48 h: Yo-Yo IR1**	Similar to baseline values
Paoli et al. (2021) [64] CT	*n* = 17 Semi-professional men 27 ± 1 years Denmark	**DURING 30 DAYS** **1. KD (ketogenic diet):** CHO < 30 g/d (10%), Prot 1.8 g/d (25–30%), Fat (65–70%) **2**. **WD (western diet):** CHO (50–55%), Prot 1.8 g/d (30%), Fat (20–25%) **→** for 30 days	**Body weight (kg)** **KD**: **Pre**: 78.19 ± 11.74 vs. **Post**: 73.98 ± 9.4 **WD: Pre:** 76.15 ± 12.03 vs. **Post**: 73.76 ± 10.13 **Body fat: KD** −7.93% vs. **WD** −4.92% **Visceral adipose tissue: KD** −16.03% vs. **WD** −7.99% **Extracellular water: KD** −3.56% vs. **WD** +0.05% **Waist circumference: KD** −4.74% vs. **WD** −1.48% **Strength and muscle mass** **Yo-Yo IR1 (m): KD** +28.04% vs. **WD** +44.62%	↓ **in both groups** **↓ KD** (*p* = 0.036) **↓ KD** (*p* = 0.0018) **↓ KD** (*p* = 0.0060) **↓ KD** (*p* = 0.0185) **↔** in both groups **↑ in both groups**

CT: clinical trial; ODS: observational-descriptive study; CHO: carbohydrates; MD: match day; TD: training day; MD-1: first day before the match; MD-2: second day before the match; MD-3: third day before the match; MD-4: fourth day before the match; Yo-Yo IR: round trip race test at increasing speed, until exhaustion; LIST: Loughborough intermittent running test; HR: Heart rate; RSA: Repeated sprint performance; Hoff: field test; ET: extra-time; CMJ: jump with countermovement; HSR: high speed running; GPS: global positioning system; CM: Central Midfielders; CD: Central Defenders; MTN: multi-ingredient supplement; H-CHO: carbohydrate-electrolyte gels; CHO-E: highly concentrated carbohydrate-electrolyte solution; **↑**: increases; **↓**: decreases; ↔: equal.

**Table 2:** Glucose levels.

Authors	Sample	Intervention	Variables	Effects
Rodriguez-Giustiniani et al. (2019) [40] CT	*n* = 18 Elite Junior Men’s Academy 18 ± 2 years UK	**BEFORE, DURING** **1. CHO (12%) + electrolytes:** 250 mL before each half of the simulated match, 60 g total CHO **2. Placebo** **→** All, breakfast 46 g CHO and 2 g/kg at lunch 2 h before the match	**Glucose at rest and after a high intensity run**	**↑ CHO,** *p* = 0.001
Harper et al. (2017) [42] CT	*n* = 15 College Men 22 ± 2 years UK	**BEFORE, DURING** **1. CHO Drink (12%) + Electrolytes** 60 g total CHO **2. Placebo (electrolytes)** **3. Water** **→** 15′ before each half of the simulated match (250 mL) **→** All, breakfast with 35 g CHO, 135′ before	**Glucose (m/mol**): **CHO-E** 4.4 vs. **Placebo** 4.2 vs. **Water** 4.2	**↑ CHO,** *p* < 0.05 vs. placebo and water, last 30′ min Glucose ↓ 27% in CHO at 60′
Krustrup et al. (2006) [43] CT	*n* = 31 men 21–33 years Denmark	**BEFORE, DURING, AFTER** **Biopsy and analysis** before, after each half, or after intense exercise after 5 sprints of 30 m with a 25 s rest. All normal standardized diet.	**Glucose**	**↔** (elevated throughout the match)
Krustrup et al. (2022) [44] CT	*n* = 20 women Professional 23 ± 4 years Denmark	**Analytical** before, after each half or after intense exercise after 5 sprints of 30 m with a 25 s rest All standardized diet	**Glucose**	↑ at the end vs. basal, *p* < 0.05
Kingsley et al. (2014) [45] CT	*n* = 14 Amateur Men 24 ± 1 years UK	**BEFORE, DURING** **1. H-CHO Drink:** 9.6% CHO + Caffeine (6 mg/kg) + Electrolytes/+ Active Gels (CHO 37.3 g + Electrolytes) **2. CHO Drink**: 5.6% CHO + electrolytes **3. Placebo** **→** 500 mL at breakfast; before each half 5.25 mL/kg and 2 gels (60 g) and 2.6 mL/kg every 15′/simulated match	**Glucose (m/mol**): **H-CHO** 6.5 vs. **CHO** 5.5 vs. **Placebo** 4.7 **Glucose at 60′**	**↑ H-CHO and CHO,** *p* < 0.001 at 45′ and 90′ vs. Placebo **↓** in all, there are no differences
Briggs et al. (2017) [47] CT	*n* = 7 Men’s Youth Premier Academy 16 ± 1 years UK	**BEFORE** **1. Rich Calorie Breakfast (RCB):** 2079 kJ, 77 g CHO, 14 g Prot and 12 g Fat **2. Usual breakfast (UB):** 1122 kJ, 39 g CHO, 10 g Prot, and 8 g Fat **→**135′ before the match, on 2 different days	**Glucose**	**↓** in both groups
Bukhari et al. (2022) [50] CT	*n* = 22 Young men 19.6 ± 1.1 years Indonesia	**DURING** **1. Dextrose (DEX):** 15 g in 150 mL (10%) **2. Sodium dextrose (Na-D):** 15 g in 150 mL (10%) + 20 mM sodium (Na) **→** previous capillary blood, 2 × 100 m run, VO _2max_ and sprint speed. Rest 15′, DEX or Na-D drink, rest 15′, capillary blood posterior. Run 2 × 100 m, VO_2max_ and sprint	**Blood glucose (mg/dl):** *-* Before: **DEX** 91 ± 11.6 vs. **Na-D** 91 ± 9.1 - After: **DEX** 136 ± 22.9 vs. **Na-D** 118 ± 21.5	**↑ DEX** vs. **Na-D** *p* < 0.05
Harper et al. (2016) [54] CT	*n* = 8 Men’s Youth Premier Academy 16 ± 1 years UK	**BEFORE, DURING, AFTER** 120′ (90′+ extra time), simulated match × 2 All drinks with electrolytes (E) before, in the middle and at 90′, 5′ before extra time gel: **placebo** or **CHO/E** 0.7 g/kg	**Glucose**	**↑** in **extra time** with CHO/E (+16%), *p* < 0.01
Mohr et al. (2023) [55] CT	*n* = 20 Men’s competition 20.1 ± 1.4 years	**→** 2 matches of 120’ separated by 3 days **→** At the beginning and after 90 and 120’ of the match	**Blood glucose (mg/dL): start**: 93 vs. **90′**: 93 vs. **ET:** 81 ± 8	**↔ start** vs. **90′** *p* > 0.05 **↓ 13% ET** vs. **90′** vs. **start** *p* < 0.05
Russell et al. (2012) [57] CT	*n* = 15 Men’s Youth Academy 18 ± 1 years UK	**BEFORE, DURING** **1. CHO (6%) + electrolytes** **2. Placebo (electrolytes)** **→** 500 mL breakfast + 3.5 mL/kg 10′ before each half and after 15, 30, 60, and 75′ of exercise (59 g CHO/h), simulated match	**Resting glucose** **Glucose between rest and 60′**	**↑ CHO-E,** *p* < 0.05 **↓ by 30% in both**
Alghannam et al. (2011) [65] CT	*n* = 6 Amateur Men 26 ± 2 years UK	**BEFORE, DURING** **1**. 0.3 g/kg **CHO** (4.8%) + 0.7 g/kg **Prot** (2.1%) **2. CHO** 1 g/kg (6.9%) isocaloric **3. Placebo** **→** 2 × 35′ with 15′ rest + run to fatigue, before and in the middle	**Blood glucose**	**Higher in CHO + Prot** at the time of fatigue, *p* < 0.05
Russell et al. (2014) [66] CT	*n* = 10 Elite Youth Men 15.6 ± 0.2 years UK	**BEFORE, DURING** **1. CHO-E: solution with 6% sucrose electrolytes** **2. Placebo with electrolytes** **→** 2 h before kick-off, before each half, every 15’ of the match (14 mL/kg/h) **→** glucose measurement every 15’ of the match	**Glucose (mmol/L)** - 30–45′: **CHO-E** 6.85 ± 0.42 vs. **placebo** 5.39 ± 0.59 - Rest: **CHO-E** 4.6 vs. **placebo** 4 - 60–75′: **CHO-E** 4.6 vs. **placebo** 4	**↑ 30% ± 12% CHO-E** at the end 1st half *p* < 0.05 **↓ 30% in both groups** at halftime *p* < 0.05 **↔** in both groups *p* > 0.05
Quiñones et al. (2022) [67] CT	*n* = 11 College Men 22 ± 3 years Canada	**BEFORE, DURING** **1. HMS: hydrothermally modified corn starch drink** (8%–0.7 g/kg/h, 2.8 kcal/kg/h) **2. DEX: dextrose drink** (8%–0.7 g/kg/h, 2.8 kcal/kg/h) **→** 2 takes: 40′ before a simulated match and at 45′ (halftime)	**Blood glucose (mg/dl) at 15′ game: HSM** 5.8 ± 0.5 vs. **DEX** 5.1 ± 0.6	**↑ HSM** vs. **DEX** *p* < 0.05.

CT: clinical trial; CHO: carbohydrates; VO2max: maximum amount of oxygen; ET: extra-time; CHO-E: highly concentrated carbohydrate-electrolyte solution; DEX: dextrose drink; **↑**: increases; **↓**: decreases; ↔: equal.

**Table 3:** Muscle glycogen levels.

Authors	Sample	Intervention	Variables	Effects
Krustrup et al. (2011) [36] CT	*n* = 7 Professional Men 27 ± 1 years Denmark	**BEFORE, AFTER** **Diet (g/kg/d): CHO** (9.5), **Proteins** (2.7) and **Fats** (0.8) (67%-20%-13% energy) + creatine. Post-match: for 5 days. → Muscle biopsy → 3 days before match: CHO 5 g/k/d (55%), 1.6 Prot	**Glycogen 0 h** **Glycogen 24 h ** **Glycogen 48 h** **Muscle pain at 0, 24, 48 and 72 h**	**↓** 57%, *p* < 0.001 vs. control **↓** 27%, *p* < 0.001 vs. control No differences, *p* = 0.096 **↑** *p* < 0.05 vs. Control
Noh et al. (2023) [41] CT	*n* = 8 College Men 21.32 ± 1.19 years South Korea	**BEFORE, DURING** **1. CHO-E: 60 g (2 × 30 g): (20 g at 12%,** ** <5 mg sodium (Na) and <0.5 g protein) (500 mL)** **→** 15′ before exercise and at rest (mixed) **2. Placebo-E** (500 mL) **→** 15′ before exercise	**Fat oxidations (g/min):** Rest: **Placebo** 0.35 ± 0.08 vs. **CHO-E** 0.47 ± 0.11 **Mean CHO oxidation (g/min): Placebo** 2.08 ± 0.26 vs. **CHO-E** 1.83 ± 0.102	**↑ CHO-E** vs. **Placebo** *p* < 0.05 **No differences** *p* > 0.05
Krustrup et al. (2006) [43] CT	*n* = 31 men 21–33 years Denmark	**BEFORE, DURING, AFTER** **Biopsy** before, after each half, or after intense exercise after 5 sprints of 30 m with a 25 s rest. All normal standardized diet	**Muscle glycogen**	**↓** *p* < 0.05 (50% mm fibers depleted, post-split)
Krustrup et al. (2022) [44] CT	*n* = 20 Professional Women 23 ± 4 years, Denmark	**Biopsy** before, after each half or after intense exercise after 5 sprints of 30 m with a 25 s rest	**Muscle glycogen**	**↓** 40% 2nd half and finish, *p* < 0.05 (69–80% mm fibers depleted post-match)
Mohr et al. (2023) [55] CT	*n* = 20 Men’s competition 20.1 ± 1.4 years	**→** 2 matches of 120’ separated by 3 days **→** Quadriceps biopsies at the beginning and after 90 and 120’ of the match	**Muscle glycogen (mmol/kg): start**: 373 ± 59 vs. **90′**: 266 ± 64 vs. **ET:** 186 ± 56	**↓ 50% ET** vs. **Start** *p* < 0.05 **↓ 29% 90′** vs. **start** *p* < 0.05
Nybo et al. (2013) [63] CT	*n* = 17 Semi-professional men 27 ± 1 years Denmark	**BEFORE, AFTER** You show muscle before, and 0, 24, 48 h post-match (**43 °C vs. control at 21 °C**) **→** Diet before and 48 h post-match (energy): 60% CHO, 15–20 Prot and 20–25 Fats	**Glycogen (0–48 h) (mmol/kg):** **Heat & Control: Pre** 488 vs. **Post 48 h** 400 **Muscle/core temperature (°C): Heat** 40.3/39.6 vs. **Control** 39.2/38.2	**↓** in both groups **↑** (1 °C) with heat
Nielsen et al. (2012) [68] CT	*n* = 7 Professional Men 27 ± 3 years Denmark	**AFTER** Muscle biopsy after high-level match and at 24, 48, 72 and 120 h (2 × 45′)	**Total glycogen volume (%):** **After Match:** 1.7 (1.5–2.0) **MD + 1: ↑** 59%; 2.7 (2.4–3.0) **MD + 2–MD + 5: ↑** 19%; (3.2–3.3)	Glycogen level remains low for a long time after a match **→** long recovery time
Gunnarsson et al. (2013) [69] CT	*n* = 16 Professional Men 24 ± 1 years Denmark	**AFTER** **1. HCP** (High CHO, Prot, Fat): 71, 21, and 8% of energy. > 8 g/kg/d **2. CONTROL (CON)** (CHO, Prot, Fats): 55, 18 and 26% **→** for 48 h after simulated match	**Muscle glycogen at the end of the match: HCP** 54% vs. **CON** 48% **Muscle glycogen resynthesis (mmol/kg)** 0–24 h: **HCP** 5.96 ± 1.02 vs. **CON** 5.94 ± 1.09 0–48 h: **HCP** 3.96 ± 0.55 vs. **CON** 4.33 ± 0.55 **Muscle glycogen according to fibers in the HCP group**	**↓** in both groups, *p* < 0.05 vs. baseline **↔** in all groups ** ↓** in type II fibers even at 48 h, *p* < 0.05 vs. basal

CT: clinical trial; CHO: carbohydrates; MD + 1: first day after the match; MD + 2: 2nd day after the match; MD + 5: 5th day after the match; ET: extra-time; CHO-E: highly concentrated carbohydrate-electrolyte solution; **↑**: increases; **↓**: decreases; ↔: equal.

**Table 4:** Fatigue.

Authors	Sample	Intervention	Variables	Effects
Noh et al. (2023) [41] CT	*n* = 8 College Men 21.32 ± 1.19 years South Korea	**BEFORE, DURING** **1. CHO-E: 60 g** (2 × 30 g)**: (20 g at 12%, <5 mg Na and <0.5 g prot) (500 mL) →** 15′ before exercise and at rest (mixed) **2. Placebo-E** (500 mL) **→** 15′ before exercise	**Perceived Exertion Index (RPE):** - Final (90′): **Placebo** 6.5 ± 0.74 vs. **CHO-E** 5.25 ± 1.04	**↓ CHO-E** vs. **Placebo** *p* < 0.05
Harper et al. (2017) [42] CT	*n* = 15 College Men 22 ± 2 years UK	**BEFORE, DURING** **1. CHO Drink (12%) + Electrolytes** 60 g Total CHO **2. Placebo (electrolytes)** **3. Water** **→** 15′ before each half of the simulated match (250 mL) **→** All, breakfast with 35 g CHO, 135′ before	**Perception of effort**	**↔**
Nacleiro et al. (2015) [48] CT	*n* = 16 Amateur Men 24 ± 3.7 years UK	**BEFORE, DURING, AFTER** **1. MTN** (53 g CHO + 14.5 g Prot + 5 g Glutamine + 1.5 g L-carnitine-L-tartrate) × 2 (total 106 g CHO) **2. CHO** 69.5 g × 2 (total 139 g) **3. Placebo** **→** before and during repeated sprint test in 90′ (4 blocks separated by 3′ rest) and 20′ after	**Perception of fatigue at the end of the test:** **MTN:** 15.9 ± 1.4 vs. **Placebo:** 17.8 ± 1.4 **MTN:** 15.9 ± 1.4 vs. **CHO:** 17.0 ± 1.9	**↓** in **MTN** *p* < 0.01 **↔**
Gough et al. (2022) [51] CT	*n* = 9 Amateur Men 21 ± 3 years UK	**DURING** **1. CHO +CAF:** 10% maltodextrin + 6 mg/kg/1 caffeine **2. CAF (caffeine):** 6 mg/kg/1 **3. CHO:** 10% maltodextrin **4. Placebo:** Water **→** 4 × 90′ soccer-specific aerobic field test (SAFT90) and two sets of repeated sprint ability tests (RSA; 6 × 6 s sprints with 24 s recovery) completed at 0′ and 75′ SAFT90 → mouthwash: 25 mL immediately before the 2nd RSA (min 75)	**Fatigue Index (FI) (%):** **1st RSA 0′ 15.40 ± 5.54** vs. **RSA 75′ 15.24 ± 5.82** **2nd RSA 0′ 11.63 ± 4.44** vs. **RSA 75′ 14.06 ± 4.89** **3rd RSA 0′ 13.62 ± 3.91** vs. **RSA 75′ 10.99 ± 2.71** **4th RSA 0′ 11.22 ± 5.29** vs. **RSA 75′ 12.77 ± 3.47**	**No differences** *p* > 0.05
Mohr et al. (2023) [55] CT	*n* = 20 Men’s competition 20.1 ± 1.4 years	**→** 2 matches of 120’ separated by 3 days **→** At the beginning and after 90 and 120’ of the match	**Fatigue:** - RSA Index (%)**: start**: 4.3 vs. **90′**: 6 vs. **ET:** 6.6 - CMJ (cm): **start**: 48 vs. **90′**: 41 vs. **ET:** 39	**↑ 1.7% RSA 90′** vs. **start** *p* < 0.01 **↑ 6.6% RSA ET** vs. **start** **↓ 19% CMJ 90’** vs. **start** **↓ 27% CMJ ET** vs. **start**
Goedecke et al. (2013) [59] CT	*n* = 22 Amateur Men 24 ± 7 years South Africa	**DURING** **1. CHO (7%) → 49 g (28 g/h if heating is included)** **2. Placebo** **→** 700 mL during simulated match (× 2, 7 days apart)	**Perception of effort** **Time to fatigue** **Time to fatigue (considering body mass)**	**↔** **↔** *p* = 0.11 **(↑ when body mass ↓)** **↑ when ↓ body mass,** *p* < 0.05 **CHO** vs. **Placebo**
Alghannam et al. (2011) [65] CT	*n* = 6 Amateur Men 26 ± 2 years UK	**BEFORE, DURING** **1. 0.3 g/kg CHO (4.8%) + 0.7 g/kg PROT (2.1%)** **2. CHO 1 g/kg (6.9%) isocaloric** **3. Placebo** **→** 2 × 35′ with 15′ rest + run to fatigue, before and in the middle	**Time to fatigue** **Perception of effort**	**↑ in CHO + Prot** *p* < 0.05 **↓ in CHO + Prot at the beginning and end,** *p* < 0.05
Spaccarotella et al. (2011) [70] CT	*n* = 8 Women’s College League *n* = 5 Men’s College League 19.5 ± 1.1 years US	**1. CHOCOLATE MILK (LCM):** 1 g CHO/kg **2. Commercial CHO + Electrolyte Drink (CE)** **→** Intake between morning and afternoon training in pre-season, 2 days	**Time to Running Fatigue** (minutes) Men + women: **LCM** 6.11 ± 5.12 vs. **CE** 5.03 ± 3.4 Men: **LCM** 8.31 ± 6.53 vs. **CE** 6.24 ± 5.03 **Perception of effort**	**↑** in LCM, no differences between beverages *p* > 0.05 **↑** in LCM *p* = 0.03 No difference between drinks

CT: clinical trial; CHO: carbohydrates; CMJ: jump with countermovement; RSA: repeated *sprint* performance; MTN: multi-ingredient supplement; CHO-E: highly concentrated carbohydrate-electrolyte solution; **↑**: increases; **↓**: decreases; ↔: equal.

**Table 5:** Cognition and gastrointestinal comfort.

Authors	Sample	Intervention	Variables	Effects
Harper et al. (2017) [42] CT	*n* = 15 College Men 22 ± 2 years UK	**BEFORE, DURING** **1. CHO Drink (12%) + Electrolytes** 60 g Total CHO **2. Placebo (electrolytes)** **3. Water** **→** 15′ before each half of the simulated match (250 mL) **→** All, breakfast with 35 g CHO, 135′ before	**Cognition** **Abdominal discomfort**	**↓** In all groups, *p* < 0.05 vs. pre-match **↑** In all groups *p* > 0.05
Briggs et al. (2017) [47] CT	*n* = 7 Men’s Premier Academy 16 ± 1 years UK	**BEFORE** **1. Rich Calorie Breakfast (RCB):** 2079 kJ, 77 g CHO, 14 g Prot and 12 g Fat **2. Usual breakfast (UB):** 1122 kJ, 39 g CHO, 10 g Prot, and 8 g Fat **→**135′ before the match, on 2 different days	**Feeling full stomach, satiety** **Abdominal discomfort**	**↑ in RCB**, *p* = 0.01 **↔**
Russell et al. (2012) [57] CT	*n* = 15 Men’s Academy 18 ± 1 years UK	**BEFORE, DURING** **1. CHO (6%) + electrolytes** **2. Placebo (electrolytes)** **→** 500 mL breakfast + 3.5 mL/kg 10′ before each half and after 15, 30, 60, and 75′ of exercise (59 g CHO/h), simulated match	**Cognitive function and motor skills**	**↑ CHO** *p* < 0.05
Quiñones et al. (2022) [67] CT	*n* = 11 College Men 22 ± 3 years Canada	**BEFORE, DURING** 1. **HMS:** hydrothermally modified corn starch drink (8%-0.7 g/kg/h, 2.8 kcal/kg/h) 2. **DEX**: dextrose drink (8%-0.7 g/kg/h, 2.8 kcal/kg/h) → 2 takes: 40′ before a simulated match and at 45′ (halftime)	**Reaction Time (ms): HSM 500** vs. **DEX 520** **Accuracy (% hits): HSM 90** vs. **DEX 90**	**↓ HSM** vs. **DEX** *p* < 0.05 **No differences** *p* > 0.05
Wolyniec et al. (2022) [71] CT	*n* = 24 Amateur Men 21.91 ± 1.30 years Poland	**DURING** 4 training sessions 40′ + 15′ rest → 35 g at 7% sugar solution (sucrose, fructose, or glucose) or 35 g xylitol (7%) during rest	**Welfare** **Tolerance** **Gastrointestinal comfort**	**↑ sucrose** vs. **xylitol** *p* > 0.05 **↓ Xylitol (osmotic diarrhea)** *p* < 0.05 **↓ sucrose** vs. **fructose, glucose** *p* < 0.05

CT: clinical trial; CHO: carbohydrates; **↑**: increases; **↓**: decreases; ↔: equal.

**Table 6:** Energy intake and availability.

Authors	Sample	Intervention	Variables	Effects
Brinkmans et al. (2019) [35] ODS	*n* = 41 Professional Men 23 ± 4 years Holland	14 days	**CHO intake (g/kg/d):** **MD** 5.1 ± 1.7; 51% vs. **TD** 3.9 ± 1.5; 45% **MD** 5.1 ± 1.7; 51% vs. **rest day** 3.7 ± 1.4; 46% **EE:** 3285 ± 354 kcal/d. vs. **EI:** MD 3144 kcal/d vs. **TD** 2637 kcal/d	**↑ MD** *p* < 0.001 MD vs. TD and rest
Morehen et al. (2022) [38] ODS	*n* = 24 Professional Women UK	12 days international campus: 4 TD, 1 MD, rest, trip, 1 TD, 1 MD, trip, 3 rest. **→**13 players USA GPS **→**24 players USA, IE	**Mean EE 12 days (kcal/d):** 2693 ± 432 **Absolute EE 4 days (kcal/d):** 2753 ± 359 **Absolute average EI 4 days:** 1923 ± 232 kcal/d - MD-4: 2172 ± 373 - MD-3: 1639 ± 285 - MD-2: 1919 ± 319 - MD-1: 1962 ± 452 **Mean relative IE CHO** (g/kg/d): - MD-4: 3.5 ± 0.9 - MD-3: 3.2 ± 1.0 - MD-2: 3.0 ± 0.7 - MD-1: 3.2 ± 1.1	**↔ EE medium 12 d** vs. **absolute 4 d** *p* > 0.05 **↓ MD-3** vs. **MD-4, MD-2 and MD-1** *p* < 0.05 **↔ MD-4** vs. **MD-2 and MD-1, MD-2** vs. **MD-1** *p* > 0.05 **Does not cover recommendations** **↔ MD-4** vs. **MD-3, MD-2** vs. **MD-1** *p* > 0.05 **Does not cover recommendations**
Dasa et al. (2023) [39] ODS	*n* = 51 Professional Women 22 ± 4 years Norway	14 days in season	**Medium EE:** 2918 ± 322 kcal (45.4 kcal/kg) **EI (kcal/d)**: **TD** 2247 ± 485 vs. **MD** 2468 ± 834 **EI CHO (w/kg/d): TD** 4.0 ± 1.3 vs. **MD** 4.5 ± 1.9 **EA (kcal/kg/d): TD** 37.9 ± 11.7 vs. **MD** 36.7 ± 17.7	**Moderate. Discrepancy of ~22%** **EE** vs. **EI** **No differences** *p* > 0.05 **↑ MD** vs. **TD** *p* < 0.05. **Does not cover recommendation** **36% MD** vs. **23% TD LEA**
Anderson et al. (2017) [72] ODS	*n* = 6 Professional Men 27 ± 3 years UK	7 days: 5 training sessions and 2 matches	**IE (kcal/d):** **MD** 3789 ± 532 vs. **TD** 2948 ± 347 **IE (kcal/d)** 3186 ± 367 vs. **EE** 3566 ± 585 **CHO intake (g/kg/d):** **MD** 6.4 ± 2.2 vs. **TD** 4.2 ± 1.4 **Exogenous CHO (g/h):** **MD** 32.3 ± 21.9 (63% gel-37% liquid) vs. **TD** 3.1 ± 4.4 (80/20%)	**↑ MD** *p* < 0.05 No differences *p* > 0.05 **↑ MD** *p* < 0.05 **↑ MD** *p* < 0.01
Rollo et al. (2021) [73] ODS	*n* = 14 Professional Men 24 ± 4 years Spain	**DURING** 4 days: 65 ± 5′ of training **1. CL (cool low):** 14 ± 7 °C, 67 ± 7% RH, low intensity. **2. CH (cool high):** 14 ± 8 °C, 69 ± 7% RH, high intensity. **3. HL (hot low):** 28 ± 1 °C, 55 ± 9% RH, low intensity. **4. HH (hot high):** 28 ± 2 °C, 55 ± 10% RH, high intensity. **→**Ad libitum access to drinks with CHO-E and water	**No. players ingested CHO-E: CL** 12 vs. **CH** 10 vs. **HL** 13 vs. **HH** 12 **Average CHO** (g/h): 15	**9 players ingested CHO-E the 4 d** vs. **3 players NO CHO-E in 2–3/4 d** **↓ CHO** *p* < 0.05 **Does not cover recommendations**
Pareja Esteban et al. (2021) [74] ODS	*n* = 20 Professional Men 19.5–31.6 years Spain	IE 7-day registration	**EE (kcal/d):** - TD moderate intensity: 3021.75 ± 192.33 - TD high intensity: 3366.40 ± 211.07 - TD very high intensity: 3704.20 ± 235.62 **Total CHO EI (g/d):** 305.07 ± 56 - TD moderate intensity: 213.6 ± 37 - TD high intensity: 361.84 ± 28 **Total EI (kcal/d):** - MD: 2940.67 ± 145 - TD moderate intensity: 2139.5 ± 142 - TD high intensity: 2205 ± 223	**↑ Very high TD** EE > **high** > **moderate** *p* < 0.05 **↑ CHO High TD** vs. **Moderate TD** *p* < 0.05 **↑ EI MD > high TD > moderate TD** *p* < 0.05
Martinho et al. (2023) [75] ODS	*n* = 25 Junior Men 14.8–15.7 years Portugal	4 days training + 1 day match	**Mean total EI 4 d (kcal/d):** 1928 ± 388. **Breakfast** 286 vs. **lunch** 491 vs. **snack** 434 vs. **dinner** 474 **EE per day:** 3568 kcal/d **Daily IE CHO:** 4 g/kg/d. **Breakfast** 0.8 vs. **snack** 1.1 vs. **dinner** 0.8	**↓ IE** vs. **EE** *p* < 0.05 **Does not cover recommendations** **↓ IE breakfast** vs. **lunch, snack and dinner** *p* < 0.05 **↓ CHO** *p* < 0.05 **Does not cover recommendations**
Anderson et al. (2017) [76] ODS	*n* = 6 Professional Men 27 ± 3 years UK	7 days: 5 training sessions + 2 matches	**IE CHO (g/kg):** **MD: Pre-match** (<1.5) vs. **Post-match** (1.0) **EI during match:** 30 g/h **IE CHO (g/kg):** breakfast 0.8; lunch 0.8; dinner 1.5; snacks (average) 0.6	**↔** Suboptimal Dinner > lunch/breakfast > snacks *p* < 0.05 Dinner vs. Rest
Macuh et al. (2023) [77] ODS	*n* = 23 Professional Men 18–35 years Slovenia	3-day pre-season food diary	**Average CHO:** 3.6 g/kg/d **EA:** 29 kcal/kg/d	**↓ CHO** *p* < 0.05 **Does not cover recommendations** **↓ EA** *p* < 0.05
Field et al. (2021) [78] ODS	*n* = 80 Professional Men ≥18 years UK	Recovery strategies	**Players who use CHO to recover** Match day vs. days before and after vs. rest days **Relationship between use and perception effectiveness**	90% 81% vs. 53% vs. 38% *p* < 0.01 attributed mostly to post-match consumption
Bettonviel et al. (2016) [79] ODS	*n* = 14 Professional Men senior (SP) 22.8 ± 3.7 years and 15 junior (YP) 17.3 ± 1.1 years Holland	For a period of 4 days (MD, MD + 1, TD, Rest)	**IE (kcal/d)** **YP** 2938 ± 465 vs. **SP** 2988 ± 583 **CHO intake (g/kg/d):** **YP** 6.0 ± 1.5 vs. **SP** 4.7 ± 0.7	**↔** **↑ YP** *p* < 0.01
Naughton et al. (2016) [80] ODS	*n* = 9 Elite Youth Men U13/14: 12.7 ± 0.6 years U15/16: 14.4 ± 0.5 years U18: 16.4 ± 0.5 years UK	7 days	**Total IE (kcal/kg/d):** **U13/14** 43.1 ± 10.3 vs. **U15/16** 32.6 ± 7.9 vs. **U18** 28.1 ± 6.8 **Relative IE CHO (g/kg/d):** **U13/14** 6 ± 1.2 vs. **U15/16** 4.7 ± 1.4 vs. **U18** 3.2 ± 1.3 **Energy distribution and CHO in separate meals**	U13/14 > U15/16 > U18, l < 0.05 U13/14 vs. both. Does not meet recommendations U13/14 > U15/16 > U18, *p* < 0.05 U13/14 vs. both and U15/16 vs. 18. Does not cover recommendations U18 lunch, dinner, and snacks > breakfast U13/14 and U15/16 snacks > lunch and dinner > breakfast
Stables et al. (2022) [81] ODS	*n* = 48 Men’s Premier Academy U12: 11.9 ± 0.1 years U13: 13.1 ± 0.2 years U14: 13.9 ± 0.1 years U15/16: 15.8 ± 0.3 years U18: 17.2 ± 0.3 years U23: 18.6 ± 1.5 years UK	IE and EI CHO before and after training during 3 days of the season	**Relative EI (kcal/kg):** PRE: **U12** 11 ± 6 vs. **U13** 11 ± 7 vs. **U15/16** 7 ± 8 vs. **U23** 7 ± 3 POST: **U12** 12 ± 6 vs. **U13** 13 ± 7 vs. **U14** 9 ± 4 **U15/16** 11 ± 3 vs. **U18** 15 ± 5 vs. **U23** 11 ± 5 **CHO EI (g/kg):** PRE: **U12** 1.5 ± 0.9 vs. **U13** 1.5 ± 1.0 vs. **U14** 0.9 ± 0.5 POST: **U12** 1.6 ± 0.8 vs. **U13** 1.6 ± 0.8 vs. **U14** 0.9 ± 0.5 **U15/16** 1.3 ± 0.6 vs. **U18** 1.6 ± 0.6 vs. **U23** 0.9 ± 0.7	**↑ U12 and U13** vs. **U15/16 and U23** *p* < 0.05. **Does not cover recommendations** **↑ U18** vs. **U14, U15/16 and U23** *p* < 0.05. **Does not cover recommendations** **↑ U12 and U13** vs. **U14, U15/16** *p* < 0.05. **Does not cover recommendations** **↑ U12 and U13** vs. **U14** *p* < 0.05. **Does not cover recommendations** **↑ U12, U13 and U15/16 and U18** vs. **U14** *p* < 0.05. **Does not cover recommendations** **↑ U12, U13 and U18** vs. **U23** *p* < 0.05. **Does not cover recommendations**
Granja et al. (2017) [82] ODS	*n* = 10 Elite Youth Men 15.8 ± 0.4 years Portugal	Dietary registration 3 days for 3 weeks. 4 days training (1 higher HTLD-load), 1 MD, and 2 days of non-training recovery within the week. At the end of the season. Only MD and HTLD were evaluated	**EI (kcal): MD** 2667 ± 170 vs. **HTLD** 2646 ± 415 **EI CHO (g/kg):** 5.2 ± 0.6 vs. **HTLD** 5.2 ± 0.9	**No differences** *p* > 0.05 **Does not cover recommendations** **↓ CHO** *p* < 0.05 **Does not cover recommendations**
Russell et al. (2011) [83] ODS	*n* = 10 Professional Youth Men 15.8 ± 0.4 years UK	Dietary registration 7 days. 1 DM, 4 TD, and 2 days of rest during the season	**EI mean (kcal):** 2.831 ± 164 **EI mean CHO (g/kg/d):** 5.9 ± 0.4	**↓** *p* < 0.05 **Does not cover recommendations** **↓ CHO** *p* < 0.05 **Does not cover recommendations**
McHaffie et al. (2023) [84] ODS	*n* = 23 Elite Junior Women 16.5 ± 0.7 years UK	10-day training camp (5 days, 3 days off and 2 days)	**Average EI:** 2505 ± 490 kcal/d **Relative EI CHO (g/kg/d):** - MD-1 1st match: 4.8 ± 1.1 - MD-1 2nd match: 4.3 ± 1.1 - MD 1st match: 4.8 ± 1.2 - MD 2nd match: 4.8 ± 1.4 **EA:** 44 ± 14 kcal/kg/d - Optimal EA (>45 kcal/kg/d) - Reduced EA (30–45 kcal/kg/d) - LEA (low) (<30 kcal/kg/d	**Does not cover recommendations** **↑ CHO MD-1** and **MD** vs. **rest of days** *p* < 0.05 8 players (38%) 12 players (57%) 1 player (5%)
Moss et al. (2020) [85] ODS	*n* = 13 Professional Women 23.7 ± 3.4 years. UK	5 days (2 rest, 1 light TD, 1 intense TD, and 1 MD)	**EI (kcal/kg): rest** 2128 ± 423 vs. **light TD** 2031 ± 548 vs. **intense TD** 2200 ± 471 vs. **MD** 2165 ± 610 **EE (kcal/d): rest** 15 ± 54 vs. **light TD** 299 ± 78 vs. **intense TD** 786 ± 159 vs. **MD** 881 ± 473 **EI CHO (g/kg): rest** 3.34 ± 0.73 vs. **light TD** 3.43 ± 0.93 vs. **intense TD** 3.05 ± 1.06 vs. **MD** 3.53 ± 1.06 **EA (kcal/kg/d): rest** 42 ± 7 vs. **light TD** 35 ± 11 vs. **intense TD** 29 ± 10 vs. **MD** 29 ± 16 - Optimal EA (>45 kcal/kg/d) - Reduced EA (30–45 kcal/kg/d) - LEA (low) (<30 kcal/kg/d)	**No differences** *p* > 0.05. **Does not cover recommendations** **↓ rest** vs. **light TD, intense TD, MD** *p* < 0.05 **↓ light TD** vs. **intense TD and MD** *p* < 0.05 **No differences** *p* > 0.05. **Does not cover recommendations** **↑ rest** vs. **light TD, intense TD, and MD** *p* < 0.05 **↑ light TD** vs. **intense TD** *p* < 0.05 15% 62% 23%
Reed et al. (2013) [86] ODS	*n* = 19 Professional women USA	Preseason	**Players with EA < 30 kcal/kg/d** **EI: 2794** ± 233 kcal/d **EI CHO: 7** ± 1 g/kg/d **EE during exercise**: 819 ± 57 kcal/d	**26.3%** due to low EI lunch *p* < 0.05 Does not cover recommendations Does not cover recommendations
	*n* = 15 Professional Women 19.23 ± 0.3 years USA	Mid-season	**Players with EA < 30 kcal/kg/d** **EA:** 35.2 ± 3.7 kcal/kg/d **EI:** 2208 ± 156 kcal/d (*p* < 0.05) **EI CHO:** 5 ± 1 g/kg/d **EE during exercise:** 642 ± 26 kcal/d	**33.3%** due to low EI in lunch and dinner, *p* < 0.05 *p* < 0.05 vs. Postseason (does not cover recommendations) **↓** *p* < 0.05 vs. Preseason (does not cover recommendations) **↓** (does not cover recommendations) **↓** Throughout the season *p* < 0.05
	*n* = 17 Professional Women USA	Postseason	**Players with EA < 30 kcal/kg/d** **EA:** 44.5 ± 3.7 kcal/kg/d **EI:** 2161 ± 143 kcal/d (*p* < 0.05) **EI CHO:** 5 ± 1 g/kg/d **EE during exercise:** 159 ± 28 kcal/d	**11.8%** Less than in preseason **↓** *p* < 0.05 vs. Preseason (does not cover recommendation) **↓** (does not cover recommendations) **↓** vs. pre and mid-season, *p* < 0.05
Magee et al. (2020) [87] ODS	*n* = 18 women College League 19.2 ± 1.1 years USA	4 days in the middle of the season	**EA** (kcal/kg/d) **EI:** 1931 ± 371 kcal/d **EI CHO:** 3.7 ± 1 g/kg/d	**Low in 67% women (high prevalence)** Low (does not cover recommendations) Low (does not cover recommendations)
Dobrowolski et al. (2019) [88] ODS	*n* = 1 Professional Women 19–24 years Poland	Start of the season	**EI:** 1476 ± 434 kcal/d **EE:** 2811 ± 493 kcal/d **EI CHO:** 3.28 ± 1.2 g/kg/d	Low (does not cover recommendations) Low (does not cover recommendations)
San Atanasio et al. (2023) [89] ODS	*n* = 19 Professional Women 19 ± 1.4 years Spain	1 week during the season: Weekdays, MD-1 and MD	**EI (kcal/d): Weekdays** 1590 ± 539 vs. **MD-1** 1790 ± 735 vs. **MD** 1657 ± 487 **EI CHO (g/kg/d): Weekdays** 2.89 ± 1.38 vs. **MD-1** 2.83 ± 1.37 vs. **MD** 2.68 ± 1.14	Does not cover recommendations **No differences** *p* > 0.05. Does not cover recommendations
Reed et al. (2014) [90] ODS	*n* = 19 Professional Women 19 ± 1 years USA	Mid-season	**Players with EA < 30 kcal/kg/d**	They consumed less post-match, *p* < 0.05
Tarnowski et al. (2022) [91] ODS	*n* = 9 Professional Women 25 ± 3 years Spain	TD and MD separated by 2 days **→**Ad libitum access to CHO-E beverages [Gatorade Low-Calorie G2 (2%) and Gatorade (6%)] and water, bananas, and gels	**EI CHO (g/h): TD** 2.0 ± 2.3 vs. **MD** 0.9 ± 1.5 **EI mean CHO (g/h): TD** 2.0 g/h vs. **MD** 0.9 g/h	**↔ TD** vs. **MD. No differences between days** *p* > 0.05 **↓ EI CHO** *p* < 0.05 Does not cover recommendations **↑ Drink 2%** vs. **6%**
Sebastiá-Rico et al. (2023) [92] ODS	*n* = 81 28 Women’s Elite Academy 53 men (13 SP, 33 YP, 35 cadets) Spain	Food Frequency Questionnaire. In season	**EI CHO**	**↓ Women** vs. **Men** *p* < 0.05
Gómez-Hixson et al. (2022) [93] ODS	*n* = 75 university students 47 men 28 women 18–23 years USA	3-day fdietary registration at the beginning of the season	**EI (kcal): Men** 2631 ± 492.8 vs. **women** 2227.7 ± 477.3 **EI CHO (g/kg): Men** 4.7 ± 1.2 vs. **women** 4.7 ± 1.5	**EI no differences between women and men CHO** *p* > 0.05. Does not cover recommendations

ODS: observational-descriptive study; CHO: carbohydrates; TD: training days; MD: match day; MD + 1: first day after the match; MD-1: first day before the match; MD-2: second day before the match; MD-3: third day before the match; MD-4: fourth day before the match; EA: energy availability (energy intake—exercise-induced energy expenditure, kcal/kg); EI: energy intake; EE: energy expenditure; GPS: global positioning system; CHO-E: highly concentrated carbohydrate-electrolyte solution; LEA: low energy availability; **↑**: increases; **↓**: decreases; ↔: equal.
